# The Aspartate-Less Receiver (ALR) Domains: Distribution, Structure and Function

**DOI:** 10.1371/journal.ppat.1004795

**Published:** 2015-04-13

**Authors:** Andrew F. Maule, David P. Wright, Joshua J. Weiner, Lanlan Han, Francis C. Peterson, Brian F. Volkman, Nicholas R. Silvaggi, Andrew T. Ulijasz

**Affiliations:** 1 Department of Biological Sciences, University of Wisconsin-Milwaukee, Milwaukee, Wisconsin, United States of America; 2 MRC Centre for Molecular Bacteriology and Infection (CMBI), Imperial College London, London, United Kingdom; 3 Department of Chemistry and Biochemistry, University of Wisconsin-Milwaukee, Milwaukee, Wisconsin, United States of America; 4 Department of Biochemistry, Medical College of Wisconsin, Milwaukee, Wisconsin, United States of America; National Jewish Medical and Research Center, UNITED STATES

## Abstract

Two-component signaling systems are ubiquitous in bacteria, Archaea and plants and play important roles in sensing and responding to environmental stimuli. To propagate a signaling response the typical system employs a sensory histidine kinase that phosphorylates a Receiver (REC) domain on a conserved aspartate (Asp) residue. Although it is known that some REC domains are missing this Asp residue, it remains unclear as to how many of these divergent REC domains exist, what their functional roles are and how they are regulated in the absence of the conserved Asp. Here we have compiled all deposited REC domains missing their phosphorylatable Asp residue, renamed here as the Aspartate-Less Receiver (ALR) domains. Our data show that ALRs are surprisingly common and are enriched for when attached to more rare effector outputs. Analysis of our informatics and the available ALR atomic structures, combined with structural, biochemical and genetic data of the ALR archetype RitR from *Streptococcus pneumoniae* presented here suggest that ALRs have reorganized their active pockets to instead take on a constitutive regulatory role or accommodate input signals other than Asp phosphorylation, while largely retaining the canonical post-phosphorylation mechanisms and dimeric interface. This work defines ALRs as an atypical REC subclass and provides insights into shared mechanisms of activation between ALR and REC domains.

## Author Summary

Two-Component Signaling (TCS) systems are a primary means by which bacteria sense their constantly changing external environment. For pathogens, these relays are key to their host survival. A typical TCS pair consists of a sensor protein, which upon an environmental stimulation will initiate a cellular response by transferring a phosphate group onto a crucial aspartate amino acid within a secondary receiver (REC) protein. However, more recently it has come to light that some REC proteins are missing key amino acids involved in this signaling relay process, including the aspartate residue required for the signaling relay, hinting that a subset of REC proteins might function in different ways. Here we use custom programing to extract all deposited Aspartate-Less Receivers (ALRs) for further examination. Surprisingly, we found that on average there are two ALRs present in every microbial species, making them a significant portion of the bacterial signaling family. Combining computational data with structural, biochemical and genetic examination of a founding member of the ALR family, Repressor of Iron Transport Regulator (RitR) from the human pathogen *Streptococcus pneumoniae*, we demonstrate that ALRs might have evolved to accommodate more diverse environmental signals, while largely retaining their time-tested ancestral post-input signaling mechanisms.

## Introduction

In a changing environment organisms must have means to effectively respond to external stimuli or perish. In bacteria, although so-called One-Component Signaling systems numerically dominate microbial genomes [1], Two-Component Signaling (TCS) systems also play crucial roles in adaptation to the changing external environment [2,3]. Classical TCS systems consist of a membrane-bound histidine kinase that upon sensing an external stimulus, autophosphorylates using ATP as a phosphate donor and then subsequently transfers the phosphate to a conserved Asp residue within a cognate Receiver (REC) domain-containing partner in the cytoplasm. REC domains adopt the flavodoxin fold, a very common (α/β)_5_ architecture with a central 5-parallell β-sheet. The typical REC active pocket structure consists of an ‘acidic triad’ that includes the phospho-accepting Asp residue at the end of the β3 strand and two other acidic amino acids within the β1-α1 loop, an ‘invariant’ lysine (Lys) residue at the end of the β5 strand and a metal ion (usually Mg^2+^) that are coordinated through hydrogen bonding [4]. Crystalline and solution structure comparisons of inactive (unphosphorylated) and active (phosphorylated) REC states have revealed a conserved signaling mechanism that centers around an equilibrium shift [5,6] and reorientation of a threonine/serine (Thr/Ser) and tyrosine/phenylalanine (Tyr/Phe) pair toward the phosphorylated Asp residue [7–11]. First described by Zhu *et al*. [12], this so-called “Y/T-coupling” results in a repositioning of the quaternary structure, usually through the α4-α5-α5 interface of the REC protein architecture, to allow the precise alignment of ionic and hydrophobic residues to form the normally observed active homodimer [13,14], although now a possible alternative dimeric interface centered around the α1-α5 face has also been described [15,16]. After these events the C-terminal Effector Domain (ED) [17], which is often a DNA-binding domain but can also take the form of many other output modules [4,18], is then freed of the physical restraint implemented by close contact with the REC domain to enable downstream signaling [4,15,19].

With a plethora of available genome sequences to sample, a second class of more divergent REC domains has emerged. These sequences typically lack one or more of the aforementioned standard ‘invariant’ features of the canonical variety, including loss of secondary structural features and key residues involved in coordination of the catalytic pocket. Some of these ‘atypical’ REC domains have diversified to accommodate signaling inputs that include binding small molecules such as antibiotics [20], and interestingly Ser-Thr phosphorylation through bacterial eukaryotic-like Ser-Thr kinases and their cognate PP2C phosphatases, which regulate crucial cell envelope-related functions and virulence in many microbes [21,22]. A key feature of many atypical REC domains is the lack of conservation of the phosphorylatable Asp residue, indicating they are regulated by input signals other than histidine phosphorylation. Described examples identified thus far come from bacterial pathogens and environmental microorganisms including RitR from *Streptococcus pneumoniae* [23,24], AmiR from *Pseudomonas aeruginosa* [17], RedZ from *Streptomyces coelicolor* [20], HP1043 from *Helicobacter pylori* [25], ChxR from *Chlamydia trachomatis* [26,27], FrzS from *Myxococcus xanthus* [28], KiaA from *Synechococcus elongatus* [29], and several have also been described in plants [30], all of which are involved in important cellular processes. As the importance of these non-canonical REC domains are becoming increasingly apparent, we wanted to know how prevalent they are in nature, if they bear functional and structural similarities to canonical REC domains, and importantly how they transduce a signal in the absence of phosphorylation.

To gain insight into these questions we first designed a custom program to extract all deposited REC domain sequences that are missing the predicted phosphorylatable Asp residue. Surprisingly, we found that these sequences comprised ~4% (or in bacteria ~2 per completed genome) of all REC domains. Given their representation, we then defined them as the Aspartate-Less Receiver (ALR) domain subclass of atypical RECs. These data reveal that although the largest category of ALR EDs consists of DNA-interacting modules, based on their ED appendages the complete ALR dataset suggests a functional consolidation into more rarely observed specialized roles such as secondary messenger signaling, RNA-binding, Ser phosphorylation and other enzymatic activities. Structural and biochemical analyses of the ALR RitR [23,24], which regulates iron and oxidative stress in the human pathogen *Streptococcus pneumoniae*, showed that in the absence of typical Asp phospho-regulation RitR likely retains the conserved Y/T-coupling mechanism of activation. Activation is shown to be prevented by an extensive ‘Hydrophobic Gate’ barrier comprised of residues within the canonical α4-β5 dimer interface. Changing the R-group in Gate residues to a methyl group (Ala) enabled RitR to actively dimerize through its α4-β5 face, bind DNA and modulate its target promoter activity (the Pneumococcal Iron Uptake (Piu) transporter) *in vivo* in the absence of an inducer. Collectively, this work presents the ALR domains and gives insight as to how they might work in the absence of typical phospho-regulatory mechanisms.

## Results and Discussion

### The ALR subfamily of REC domains

To reveal the extent to which the REC domain family was missing the conserved phospho-accepting Asp, we downloaded the available (103,233) REC sequences from the Pfam database, and from this used a series of custom programs to extract a subset of 3,484 sequences lacking the phospho-regulated Asp residue. When redundant sequences were removed 74,816 unique REC-containing proteins were identified, 2,976 of which were missing or had substitutions at the conserved Asp position and indicated that as much as 4% of REC domains *do not* possess this conserved phosphorylatable residue. Given the frequency of these substitutions we renamed this subset of REC-type sequences Aspartate-Less Receivers (or ALRs). The full dataset of ALR sequences and their accession numbers are given in S5 Table. ALR ED architectures, their accompanying Pfam ED accession numbers and phyla associated with these specific EDs are listed in S6 Table.

### Analysis of ALR substitutions

**Fig 1:**
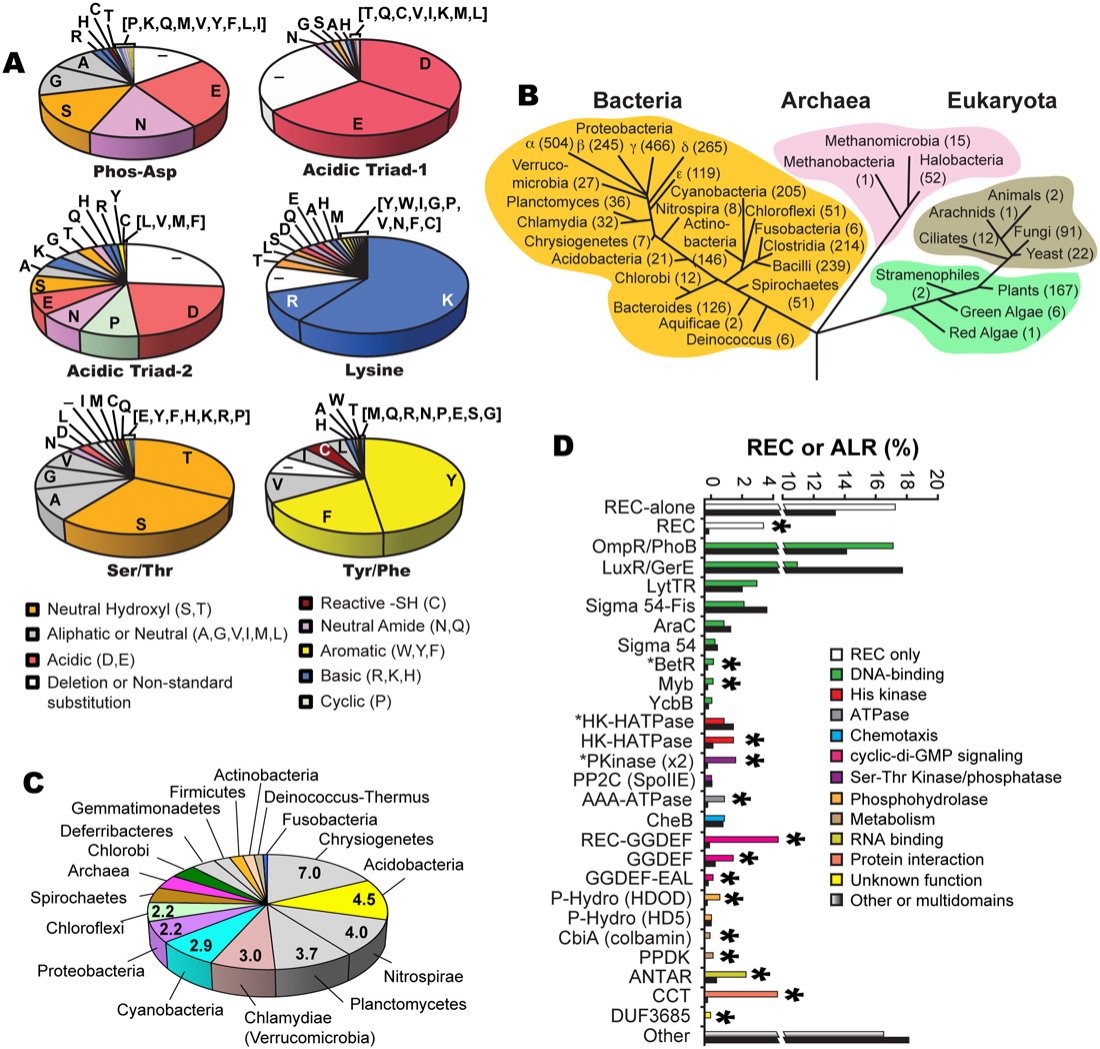
ALR statistics and phylogeny. (**a**) Frequency of amino acid substitutions within six key ‘invariant’ REC residues in ALR sequences: the (now changed in ALRs) conserved histidine kinase phosphorylated aspartate residue position that defines the ALR subfamily (Phospho-Asp), acidic triad residue-1 (Glu9 in RitR) and acid triad residue-2 (Lys10 in RitR) that normally help coordinate the metal ion active pocket, the Tyrosine/Phenylalanine (Tyr/Phe, Tyr100 in RitR) and Threonine/Serine (Thr/Ser, Asp81 in RitR) that make up the Y/T-coupling system, and the conserved pocket Lys (Lys103 in RitR). Notice that where catalytic active pocket Asp/Lys residues have often been changed in ALR sequences (top panel), the T/Y-coupling residues generally remain conserved (bottom panel). This trend in conservation is also observed for the acidic triad-1 and the universally conserved pocket Lys residue (Lys103 in RitR), but not for acidic triad-2. (**b**) Taxonomic distribution of ALR sequences. The number of ALRs discovered in the given class or phylum is shown in parentheses. (**c**) Distribution of the average number of ALR sequences per completed genome by phyla. (**d**) Bar graph of the percentage contribution of a given Effector Domain (ED) within total REC sequences (shown as black bars) and ALR sequences only (shown as non-black bars). An asterisk above the bars indicates that ALRs are enriched for the ED by over 50% within the ALR population compared to their representation within typical REC sequence populations. An asterisk in front of the ED name indicates that the ALR or REC domain is (unusually) C-terminal to the ED sequence.

Examination of our ALR dataset revealed several substitutions within predicted acidic triad positions, which includes the phosphorylatable Asp residue (phospho-Asp). In typical REC sequences these three acidic residues facilitate the phospho-transfer reaction and then help to coordinate the newly formed phosphorylated active pocket. We found that the former phosphorylated Asp position is most frequently substituted with a Glu (26%; Fig 1a). In typical REC sequences when the phospho-Asp is replaced with Glu this can result in a constitutive phosphate-independent activation [31–33]. However, often Asp to Glu substitutions alone are not sufficient to produce such an effect, and therefore must be combined with other mutations to result in constitutive activity (*e*.*g*. CheY [34], PhoB [35] and Spo0A [36]). Similar to canonical REC domains, ALR constitutive activity might also be enabled by a change in the phosphorylatable Asp to Glu along with other key functional residues. For example in the case of ChxR, an ALR from *Chlamydia trachomatis* that contains a Glu in this position, when tested *in vitro* substitutions with Asp or Ala alone were not sufficient to abrogate its observed constitutive DNA-binding activity [37]. After Glu, the next most frequent substitutions at the conserved phospho-Asp are Asn (16%), Ser (15%), Gly (11%) and Ala (9%) (Fig 1a), all of which have a representative characterized ALR and accompanying atomic structure (except for Gly; Figs 1a and S2a; S3 Table).

The remaining 23% of ALR phospho-Asp substitutions are far less frequent, some of which would be predicted to significantly change the typical REC active pocket hydrogen-bonding network. For example, we found 21 ALR sequences with a Pro substitution and another 23 sequences with either a Phe or Tyr aromatic. It is also worth noting that a substantial number of deletions in ALRs are observed in and around the former phospho-Asp position and other pocket residues, which would be predicted to result in changes to the typical REC domain pocket and/or overall structure (Fig 1a). Although only two examples thus far of an ALR display an absent portion of its secondary structure within the pocket region (ChxR [37] and HP1043 [38]), our data presented here suggest that such structural features might be more common in the ALR domain family than in typical RECs. Other more rarely used residues at the former phospho-Asp position include the positively charged (basic) amino acids of Arg, Lys and His, and when combined were found to constitute 4% of ALR phospho-Asp substitutions. One possibility is that such an alteration in charge or a similar change in *any* of the acidic triad residues might suffice to replace the missing active pocket metal ion (yet to be identified in any ALR). The only amino acid that we did not find in place of the phospho-Asp was Trp, presumably due to the large steric clashes and instability that would likely ensue on the conserved REC three-dimensional architecture. In fact, only one Trp substitution was discovered among all three acid triad residues in ALRs. In general other hydrophobic residues (Val, Ile, Leu, and Met) were also rarely observed in place of any of the acidic triad amino acids (Fig 1a). One exception to this rule appears to be Ala substitutions, where its single methyl side chain might disrupt the overall pocket structure to a lesser extent. In support, the described ALR FrzS structure from *M*. *xanthus* takes on a typical REC (α/β)_5_ fold where the phospho-Asp has been substituted with an Ala residue (ref. [28] and S2a Fig).

Another noteworthy observation was that the N-terminal acidic triad residue (referred to here on as “acidic triad-1”, in RitR it is coordinate Glu9) is generally retained in ALRs (65%), whereas the second acid triad residue (referred to here on as “acidic triad-2”, in RitR it is coordinate Lys10) is only an acidic residue in 29% of ALR sequences (Fig 1a). Furthermore, the conserved Lys that helps coordinate the typical active pocket in REC domains was also largely retained in our ALR dataset (70% of sequences were basic at this position, in RitR it is coordinate Lys103) (Fig 1a), suggesting that this residue and at least one acidic triad residue (more often acidic triad-1) are maintained in ALR sequences. Whether these conservations are present to maintain structural integrity, protein function or both is at present unclear.

We also examined the retention of Y/T-coupling residues in ALRs. Based on sequence alignment, available structural information and computational methods, Figs 1a, S1, S2, S3 and S3 Table collectively show that in the majority of cases Tyr/Phe (Y/F) and Thr/Ser (S/T) residues are present in ALRs at the expected positions (66% and 61% of the time, respectively). We also calculated that both a Y/F and S/T residue were present within the *same* ALR domain 44.5% of the time, which indicates that in ALRs the pairing of these residues is important, but to a lesser extent than canonical REC domains. Interestingly, if an S/T residue is present in an ALR sequence (61%) then a Y/F is enriched for and appears 77% of the time, whereas if a Y/F is present (66%) then an S/T residue appears at the same rate of 66% (S3 Fig). We also observe that if one residue of the Y/T pair is present, then the other appears with a greater frequency than observed in the total ALR population (for Y/F residues 11% more, and for S/T residues 5% more; S3 Fig). Combined, these observations suggest that in ALRs there is selective pressure to retain Y/T coupling residues, and to a certain extent (44.5%) to keep them together in the same ALR. Importantly, our data also show that in ALRs it is more important to retain the Y/F residue than the S/T.

One explanation for these data could be that to accommodate alternative functions assigned to the sometimes drastically changed ALR “active pockets”, the S/T residue might have to change more frequently as it (but not the Y/T partner) is actually part of the altered catalytic core. Thus the S/T residue could be subjected to a greater evolutionary pressure to accommodate changes in the active pocket specific to a particular ALR function (*e*.*g*. atomistic coordination of a bound small molecule). We observe that if one of the Y/T residues is present but not the other, then the substitution tends to be hydrophobic (*e*.*g*. Val, Ile, Ala, Leu), and is infrequently charged (RitR is one exception to this rule as it has an Asp residue in place of the Ser/Thr). The reason for these preferences remains to be determined. Taken together our results shown here allude to selective pressure in ALRs to retain Y/T-coupling residues, whose exact roles in signaling will likely have to be determined on an individual experimental basis.

### Potential for other regulatory posttranslational modifications (PTMs)

Intriguingly, we noticed that many of the substitutions in ALRs are capable of receiving PTMs other than Asp phosphorylation. The most common ALR substitution Glu (Fig 1a) has been shown as phosphorylatable [39], however to the best of our knowledge there are no known examples of such modifications contributing to bacterial signaling. A more likely Glu-specific PTM would be methylation, as this modification has already been convincingly shown to alter bacterial protein signaling function [40]. The next most common substitution, Asn (as seen in RitR; Fig 1a), has the potential to become modified by deamidation to a phosphorylatable Asp. Indeed it has been demonstrated that a REC domain harboring an Asn at the phosphorylatable position can undergo rapid reversion back to Asp [41]. How prevalent this reaction is in Asn-harboring ALRs, and if there are functional consequences of such chemical reversions has yet to be explored with RitR and other ALRs.

We also noticed that the third most common ALR substitution at the conserved phospho-Asp site is Ser, and when combined with Thr and Tyr replacements these phosphorylatable residues together constitute 12% (556 sequences) of all identified ALRs (Fig 1a). These amino acids are all well known to hold potential for phospho-regulation by bacterial Ser-Thr [42] and Tyr [43] kinases. Additionally 63 ALR sequences harbor an Arg at the typical REC phospho-Asp position, an amino acid that also has the potential for phospho-regulation in prokaryotes (by arginine-specific kinases and their cognate phosphatases [44,45]). We also noted that the seventh most common substitution at the phospho-Asp position was Cys, a residue capable of sensing environmental changes in the cellular redox state [46].

**Fig 2:**
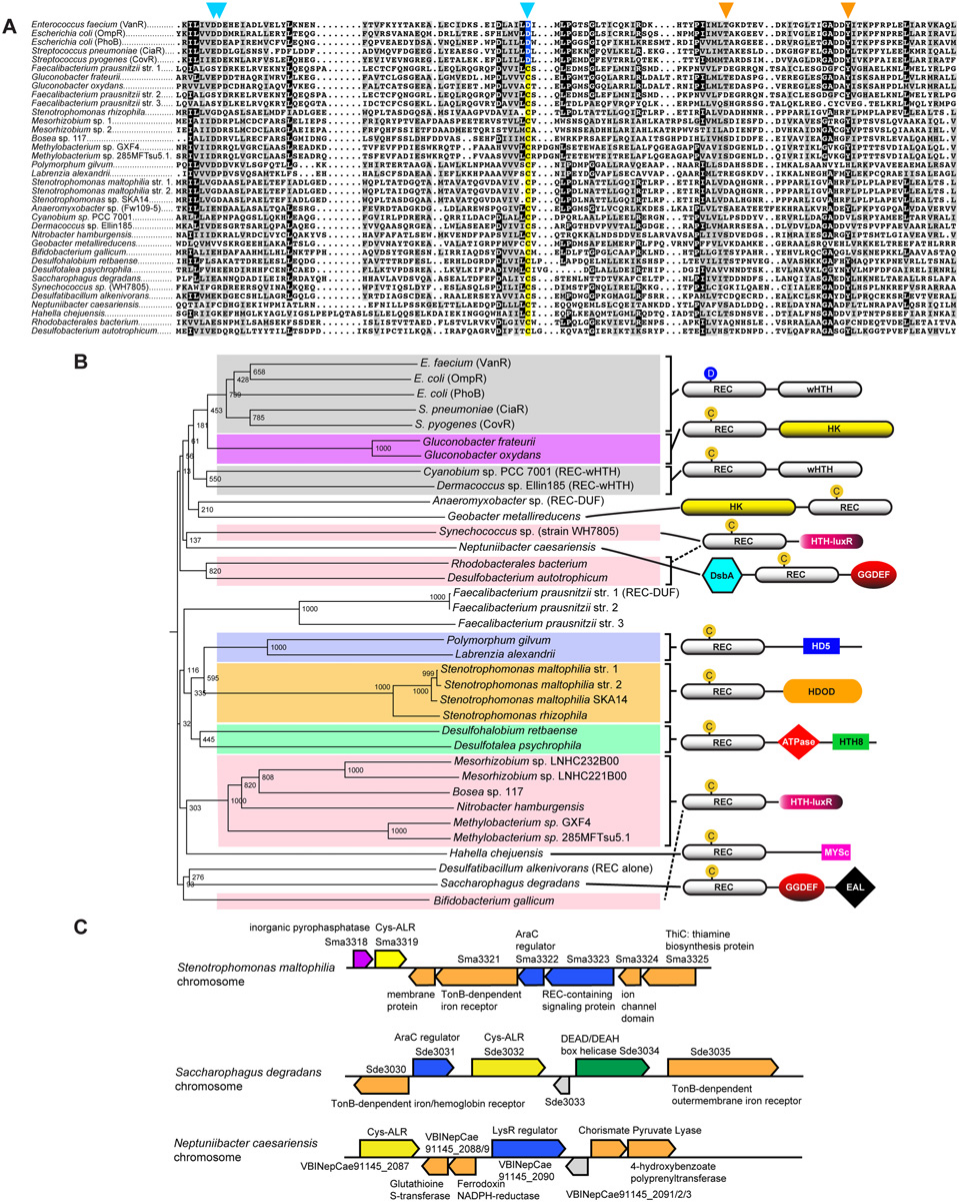
Cys-ALRs. (**a**) Alignment of the 26 extracted ALR domains that contain a cysteine residue in place of the typical phosphorylated Asp seen in canonical REC domain sequences (colored yellow). For comparison, Asp-containing REC domains VanR, OmpR, PhoB, CiaR and CovR were included and their conserved phosphorylatable Asp residue (colored blue). The ALR sequences were imported in FASTA format into Clustal X 2.1 [82]. The alignment was then uploaded into MacBoxShade 2.15 (Institute of Animal Health, Pirbright, UK) for visual representation. (**b**) Phylogenetic tree of Cys-ALRs shown in (a). Related clades are grouped by color and a schematic representation of their domain architecture is shown on the right. Posterior probabilities are shown at the branch points. The circle with a “C” or “D” indicates a Cys or Asp amino acid, respectively, located at the phospho-Asp position. Domain architecture abbreviations are as follows: REC, receiver; HK, histidine kinase; HTH, helix-turn-helix; wHTH, winged helix-turn-helix; DsbA, bacterial disulfide oxidoreductase; GGDEF, cyclic-di-GMP; EAL, diguanylate phosphodiesterase; HDOD and HD5, phosphohydrolase; HTH-luxR, luxR family of bacterial transcription factors; MYSc, myocin domain; DUF, domain of unknown function. The alignment was generated using Clustal X 2.1 [82] and uploaded for phylogenetic display into Archaeopteryx [83].

The 26 identified ALR sequences with a Cys substitution (Cys-ALRs) were further examined by generating an alignment (Fig 2a) and accompanying phylogenetic tree (Fig 2b). Results revealed a distribution among both pathogenic and environmental microbes, many of which require strict oxygen conditions for growth (*e*.*g*. *Neptuniibacter caesariensis*, *Bifidobacterium gallicum* and *Geobacter metallireducens*). Cys-ALR EDs were found to cluster into defined outputs and include a DsbA module from the marine aerobe *N*. *caesariensis* (Fig 2b), a domain which can act as a disulfide oxidoreductase to ensure proper folding of proteins, especially excreted toxins and virulence factors [47]. Examination of the *N*. *caesariensis* genome revealed its encoded Cys-ALR to be adjacent to an operon containing glutathione S-transferase and a predicted NADPH ferrodoxin, enzymes that Dsb family members are often associated with [47] (S7 Fig). Two other predicted genes encoding for Cys-ALRs from the marine bacteria *Saccharophagus degradans* and human pathogen *Stenotrophomonas maltophilia* were found adjacent to operons encoding iron/heme uptake systems (S7 Fig).

At present PTMs other than histidine kinase phosphorylation have not been described at the conserved REC domain phospho-Asp position. However, there is a plethora of literature describing alternative PTM regulation at other REC and associated ED residues by bacterial kinases [42,43,45,48], as well as by Cys-mediated oxidation [46]. To the best of our knowledge the only example thus far of an atypical REC or ALR domain-containing protein being regulated through PTM modification at any residue is RitR (Ser phosphorylation of the DNA-binding domain [24]). Although speculative, it is exciting to think that some ALRs might be regulated at their former phospho-Asp site by other PTMs (*e*.*g*. Ser, Thr, Tyr, Arg phosphorylation or oxidation), whereby the newly-formed modified amino acid would be accommodated by a novel restructured ALR catalytic pocket. Future work will be required to make a definitive verdict on this subject.

### ALR phylogenetic distribution

The phylogenetic numeric distribution of ALRs spanning all three domains of life is depicted in Fig 1b, and also displayed in Fig 1c as the average number of ALRs per Completed Genome (CG) categorized by phyla. Expanded bioinformatic statistics of typical REC domains versus that of ALRs are displayed in S1 Table, and ALR EDs and their association with specific phyla are given in S6 Table. For bacteria we found on average that any given CG possesses 2 ALR sequences, making ALRs a significant portion of the REC-like sequences in prokaryotes. In fact, only 5 out of the 45 known bacterial classes lacked ALRs (the *Mollicutes*, *Thermotogae*, *Dictyoglomia*, *Elusimicrobia* and *Synergistia*; S1 Table). Although ALRs have thus far been predominantly characterized in pathogens, our data show they are numerically dominated by environmental bacteria, where greater selective pressures might be responsible for driving REC divergence to accommodate new input signals. We found that the highest average number of ALRs per CG were in the arsenic utilizing *Chrystiogenetes* (7 per CG), followed by the largely unstudied and mainly soil-dwelling *Acidobacteria*, the budding *Plantomycetacia*, the nitrite-oxidizing *Nitrosprira* and the phylum of *Verrucomicrobia* (*Clamydiae*). Photosynthetic bacteria also were enriched for ALRs including the oxygenic photosynthetic *Cyanobacteria* and the *Chloroflexi* green non-sulfur bacteria (Fig 1c and S1 Table). In general, the expansive phylum of *Proteobacteria* was found to contain an average of 2.2 ALRs per CG. However, the class of δ-*proteobacteria*, which is largely composed of the related pathogenic species of *Helicobacter* and *Campylobacter*, was the most highly enriched in this taxon possessing approximately 5 ALRs per CG (S1 Table).

ALRs were found to comprise as much as 10% of the total REC sequences in Archaea, with the classes of *Halobacteria* and *Methanomicrobia* containing the highest numbers (Fig 1b and S1 Table). Eukaryotes were also enriched in ALR sequences, which were found in yeast, fungi, ciliated protozoa, land plants and both green and red algae (Fig 1b and S1 Table), but were most abundant in the *Eurotiomycetes* class of fungi (58 ALRs), the plant phylum of *Streptophyta* that includes vascular plants (88 ALRs), and finally the highly-evolved *Liliopsida* (or lilly) class of flowering plants (75 ALRs). A surprising finding was the presence of REC domains and some ALRs in ticks (*Arachnida*, 2 RECs, 1 ALR), and the primitive invertebrate marine taxa of *Anthozoa* (mainly sea urchins, 17 RECs, 1 ALR) and *Placozoa* (4 RECs, 1 ALR), Placozoa being the most basal forms of invertebrates known [49]. As far as we are aware REC sequences and TCS systems have yet to be described in insects and animals. These sequences could be artefacts of bacterial contamination and/or endosymbionts [49], but given the potential for expansion of TCS into the animal kingdom further investigation is warranted.

### ALR Effector Domains (EDs)

To shed light on the enrichment of ALR ED sequences and how they might correlate with specific taxa, we extracted all ED statistics associated with canonical RECs and ALRs and cross-referenced these results with their taxonomic distribution. Results shown in Fig 1d and S2 Table indicate that the majority (53%) of canonical REC sequences are either ‘stand alone’ REC domains, or possess C-terminal DNA-binding domain extensions, with the largest portion being OmpR/PhoB-type transcription factors such as RitR [50], followed by the LuxR/GerE family. For most DNA-binding effectors, their percentage association with ALRs roughly paralleled the percentage associated with total REC sequences (green versus black bars in Fig 1d). On the other hand, the less commonly seen DNA-binding EDs of YcbB (or GlnL) that possesses a novel helix-turn-helix motif, Myb domains that are dominant in plants, and the *β*-*proteobacterial* transcriptional regulator (BetR) family whose function(s) is currently unknown, were all considerably enriched in ALRs (Fig 1d and S2 Table). The remaining 47% of ED sequences are diverse in their functional roles and phylogenetic distribution (a complete listing can be seen in S6 Table). In particular, our data show that ALRs were highly enriched for when attached to RNA binding (ANTAR) motifs, cyclic-di GMP (GGDEF) signaling domains, the Constans-Constans–like TOC1 (CCT) domains involved in controlling plant circadian rhythms, and modules containing both Ser-Thr kinase and phosphatase domains [17,18,30] (Fig 1d and S2 Table). Also highly associated with ALRs were several enzymatic EDs that include pyruvate phosphate dikinases involved in plant C4 carbon metabolism, and HDOD phosphohydrolase domains whose exact functional role(s) is currently unknown [18].

These findings reveal that although ALRs exist in greater overall numbers within the more common ED families (*e*.*g*. OmpR-type and LysR), they represent a larger percentage of more rare and less studied REC-associated effector outputs. To further illustrate this point the OmpR-type EDs are associated with the highest raw number of ALRs (609 sequences), yet this figure only constitutes 2% of all REC-[OmpR/PhoB winged helix-turn-helix] architectures. In contrast, out of only 24 REC-CbiA (colbamin binding domain) architectures known to exist, 11 are ALRs (or 46%; Fig 1d; S2 Table).

### Crystal structure and analysis of the ALR RitR

To the best of our knowledge, there are currently five available functionally-characterized prokaryotic ALR structures: (i) AmiR from *Pseudomonas aeruginosa* [17], (ii) HP1043 from *Helicobacter pylori* [25], (iii) ChxR from *Chlamydia trachomatis* [26,27], (iv) FrzS from *Myxococcus xanthus* [28] and (v) KiaA from *Synechococcus elongatus* [29]. However, our computational searches revealed an additional 5 unpublished and uncharacterized ALR structures that have been deposited in the RCSB protein databank (www.rcsb.org; PDB IDs 2B4A, 2QZJ, 2ZAY, 3HV2, and 3KTO). For comparison available ALR structures and their known attributes are listed in S3 Table, and an alignment of these sequences and structural comparisons are shown in S2 Fig. To add to the understanding of how ALRs are able to function in the absence of a phospho-Asp signal, and to decipher if RitR parallels these previously solved ALR as well as canonical REC structures, we obtained a crystal structure to 1.6 Å resolution of the ALR (REC) domain of RitR (RitR_ALR;_ PDB ID 4LZL; Table 1), responsible for regulating oxidative stress and iron uptake in the important human pathogen *S*. *pneumoniae* [23,24].

**Table 1:** Crystallographic data collection and model refinement statistics.

Data Collection
Wavelength (Å)	0.97931
Space group	P1
Unit cell	a = 28.46, b = 33.84, c = 34.96 Å α = 92.65, β = 103.71, γ = 97.53°
Resolution range (Å)^a^	27.35–1.55 (1.60–1.55)
Reflections observed	50,967 (7,357)
Unique reflections	17,289 (2,488)
Completeness (%)	94.63 (93.38)
Mean I/σ(I)	12.08 (6.61)
Multiplicity	2.9 (3.0)
R_symm_ ^b^	0.054 (0.090)
Wilson B-factor	11.62
Model Refinement	
R_cryst_ / R_free_	0.136 (0.141) / 0.157 (0.152)
Protein residues	123
Number of atoms (excluding H)	
Macromolecule	1102
Ligands (glycerol)	15
Solvent	184
RMS deviations from ideality	
Bonds (Å)	0.009
Angles (°)	1.17
Ramachandran favored / outliers (%)	98.0 / 0.0
Clashscore^c^	11.07
Average B-factors (Å^2^)^d^	
All atoms	12.3
Macromolecule	10.0
Solvent	25.6

^a^Values in parentheses apply to the high-resolution shell indicated in the resolution row.

^b^R = Σ(||Fobs|-scale*|Fcalc||) / Σ |Fobs|.

^c^Number of close interatomic contacts per 1000 atoms.

^d^Isotropic equivalent B factors, including contribution from TLS refinement.

### Overall RitR structure

**Fig 3:**
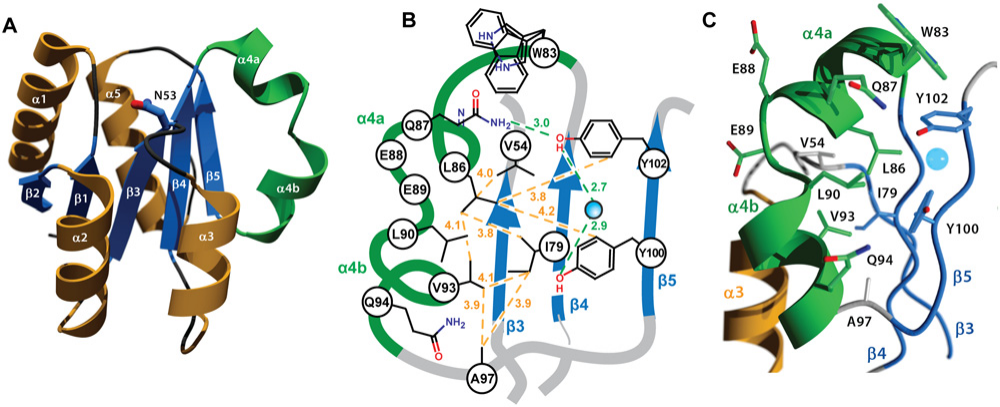
Crystal structure of the RitR REC domain. (**a**) Cartoon representation of RitR_ALR_ with helices α1- α3 and α5 colored orange, the unusual α4 helix colored green, and the β-strands colored blue. The equivalent of the phospho-modified Asp residue found in typical REC domains, RitR coordinate Asn53, is shown as ball-and-stick with blue carbon and red oxygen atoms. (**b**) Schematic view showing the pattern of RitR van der Waals interactions (yellow dotted lines) and hydrogen-bonding network (green dotted lines) in the dimer / Gate region of the structure. (**c**) Close-up of the kinked α4 helix (in green) and surrounding residues. The blue sphere is a water molecule.

As implied by its crystallization in the P1 space group, which lacks crystallographic symmetry elements, RitR_ALR_ is monomeric and suggests that this structure could represent an inactive state of the protein [4]. The overall fold of the RitR structure shares the (α/β)_5_ topology common to the OmpR/PhoB family of response regulators [4,50,51] (Fig 3a). One notable exception is a defined kink in the α4 helix, which we have subdivided into α4a that interacts with the would-be catalytic pocket in RitR, and α4b that likely interacts with helix 8 of the DBD to preclude its association with DNA [15] (Fig 3b and 3c). A search of available ALR and typical REC atomic structures revealed only two others that exhibit a similar α4 helical bend: PhoP from *Mycobacterium tuberculosis* [52] (PDB ID 3r0j) and DrrD from *Thermotoga maritima* [53] (PDB ID 1KGS).

### The altered catalytic pocket of RitR

**Fig 4:**
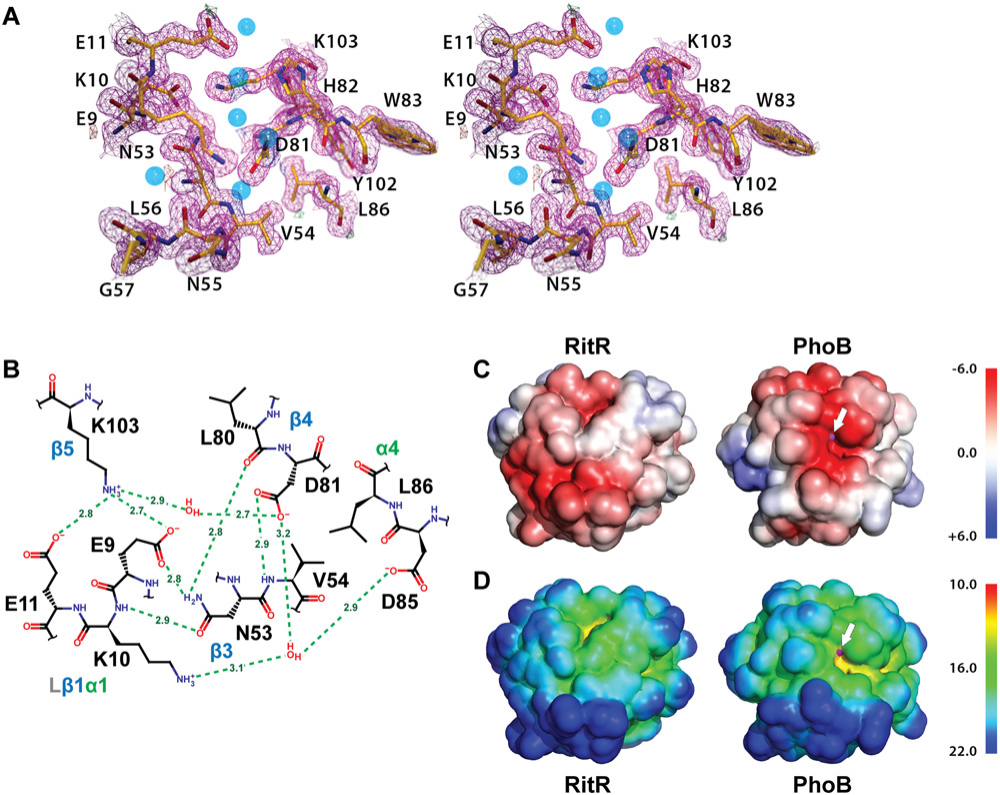
Structure of the RitR ‘active’ pocket. (**a**) Stereoview of the electron density in the RitR active site (magenta mesh) contoured at 1.5 σ. Water molecules are shown as blue spheres. Notice the lack of a metal ion in the typical metal-binding site near Glu9. (**b**) Schematic view of the RitR REC ‘active site’ showing predicted hydrogen-bonding interactions (green dotted lines) with distances in Angstroms (Å). (**c**) Comparison of the vacuum electrostatic surface potentials of RitR_ALR_, *left*, and the PhoB REC domain, *right*. The Mg^2+^ site in PhoB is indicated by a magenta sphere, which can be seen protruding slightly through the surface (denoted by the white arrow). (**d**) Comparison of the surfaces of the RitR and PhoB REC domains colored by distance from the center of mass of each protein. Not only is the electronegative environment in the metal-binding site of PhoB lost in RitR, the cleft that normally holds the metal ion (yellow region near the white arrow) is missing as well. Figure generated using PyMol (Version 1.4.1, Schrödinger, LLC).

Similar to other ALRs and atypical REC sequences, the would-be “active site” of RitR harbors several divergent substitutions relative to classical REC domains (Figs 1a, S1 and S2), all of which gave well-defined electron density for analysis (Fig 4a). First, the phosphorylatable Asp position in RitR is substituted with asparagine (Asn53), thus defining it as a *bona fide* ALR. Although Asn53 is in approximately the same position as its more canonical Asp-containing counterparts, the conformation differs by a roughly 90° rotation of the χ1 angle where it now hydrogen bonds to acidic triad-1 (Glu9; Figs 3b and S2), which in typical REC domains binds the Mg^2+^ ion required for the phospho-transfer. When compared with other ALR structures we see that the acid triad-1 position and conserved would-be active pocket Lys (RitR coordinate Lys103) almost without exception make electrostatic interactions to presumably help stabilize the ALR ‘active’ pocket, with the residue that replaces the phospho-Asp site also participating when side chain hydrogen bonding is possible (S2 Fig). Conversely, the acidic triad-2 position side chain only forms a potential hydrogen bond with other ALR acid triad residues in the case of AmiR (S2 Fig). These observations suggest that ALRs have largely retained an acidic residue at the acidic triad-1 position (66%) and a basic residue at the conserved Lys position (70%) to maintain the overall structure of a typical REC domain, while the acid triad-2 remains much more variable (only 29% conserved in ALRs; Fig 1a).

Of note, in many ALRs such as RitR, HP1043 and 3KTO acidic triad-2 is replaced by a positively charged (or basic) residue, for example in RitR by Lys10 (Figs 4a and 4b and S2). This normally invariant position of the classical REC domain, located between the β1 strand and α1 helix (Lβ1α1), is crucial in coordinating the active pocket metal and phospho-Asp residue in typical REC sequences. One hypothesis is that such a change to a positive charge might act to take the place of the normally present positively-charged Mg^2+^ ion, and therefore serve to maintain the structural integrity of the overall REC/ALR fold. In RitR we see that the Lys substitution at acidic triad-2 dramatically alters the position of the entire Lβ1α1 loop relative to typical REC domains such that the cleft, which would normally receive the metal ion, collapses and interrupts what would usually be in typical REC structures a continuous electronegative surface (Fig 4c and 4d).

Whatever the reason for these observed changes, our computational and structural data presented here point to ALRs retaining at least one of the two remaining acidic triad residues (usually acidic triad-1), and the conserved Lys-Pro motif, which together help to maintain the classical three-dimensional REC structure in the absence of the usually bound metal ion and typical Asp phosphorylation. Conversely, variation seen within the other two acidic triad residues (the changed phospho-Asp position and acidic triad-2) might be tailored to individual ALR mechanisms.

### Y/T-coupling and the ‘Hydrophobic Gate’

A distinguishing feature of the RitR structure is the α4 helix, which is broken at residues 88–90 into two smaller helices (α4a and α4b; Fig 3a–3c). A careful examination of the available REC structures reveals variations in this region from a straight helical extension. However, few REC α4 structures are as profusely interrupted as the RitR α4, where the measured angle between the helical axes of α4a and α4b is approximately 100°. Situated on the α4 are three hydrophobic residues (Leu86, Leu90, and Val93) that pack against the central β-sheet and make van der Waals contacts with Val54 (Lβ3α3), Ile79 (β4), Ala97 (Lα4β5), Tyr100 (β5) and Tyr102 (β5) to form what we name here the ‘Hydrophobic Gate’ (or ‘Gate’ residues; Fig 3b and 3c).

The Gate creates an obvious mechanical barrier to impede the reorientation of Tyr100 (*i*.*e*. Y/T-coupling), which in REC domains usually results in α4- β5- α5 dimer association and DNA binding [10,11]. In RitR we noticed that the position of residue Leu90 appeared especially important in that it directly blocks Tyr100 from entering the Gate and also holds the helix 4 kink together through a hydrogen bond (2.7 Å) to the carbonyl of Glu87. As a result, the side chain of the Tyr100 dimerization (Y/T-coupling) trigger is oriented outward to extend into the solvent—away from the α4-β5- α5 face of the protein as observed in the typical monomeric, inactive REC conformation [7,9]. This positioning might explain why in the case of RitR we observe a predominantly monomeric state in both crystalline and solution environments (see below). The outward-facing Tyr100 rotomer is also enforced by Gate residue Leu86, which in RitR occupies the space normally filled by the Y/T-coupling Thr or Ser residue in the unphosphorylated state. Instead, the equivalent of this residue in RitR (Asp81) orients itself towards Asn53, which is reminiscent of the active or inward-facing rotomer when typical REC domains are phosphorylated. Thus in our RitR structure Y/T-coupling is effectively trapped in an ‘uncoupled’ intermediate state, whereby Asp81 is in the “in” orientation and Tyr100 in the “out” orientation.

**Fig 5:**
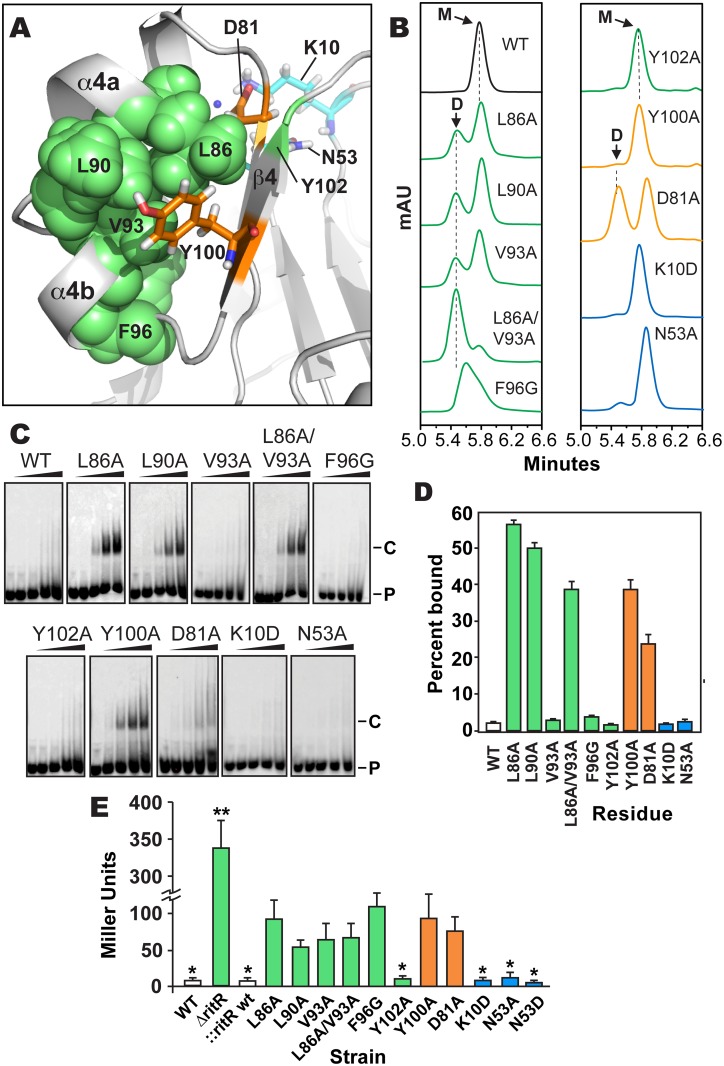
Analysis of RitR mutations. (**a**) Cartoon representation of the RitR_ALR_ atomic structure depicting important Gate residues (shown in green), the conserved Tyr100 and Asp81 Y/T-coupling residues (shown in orange) and acidic triad residues (shown in cyan). (**b**) SEC of RitR_FL_ variants (see S6 Fig for protein purity). mAU, milli Absorbance Units; WT, Wild-type RitR_FL_ protein; D, Dimeric form of RitR_FL_; M, Monomeric form of RitR_FL_. (**c**) EMSA shifts of RitR_FL_ variants in the presence of HEX-labeled BS2 33-mer double-stranded DNA oligomer at 0, 0.22, 0.66, 2.2 and 6.6 μM concentrations (left to right). P, Hex-BS2 DNA Probe; C, RitR-(HEX-BS2 DNA) shifted Complex. (**d**) EMSA quantification of RitR_FL_ variants (2.2 μM) binding to HEX-BS2 DNA. Values represent mean +/- SEM, n = 3. (**e**) Effect of RitR mutations on Piu promoter activity as measured by β-galactosidase levels (in Miller units). One asterisk indicates a P value of <0.05, and two asterisks a P value of <0.01 as determined by Student’s t-test. Error bars represent mean +/- SEM. t-test comparisons were made using the Y100A mutant as a reference.

A survey of the available ALR structures reveals that the RitR Tyr100 outward-facing rotomer is not the norm. In fact, all but one currently available ALR structure exhibits an inward-facing conformation, which could explain why several ALR structures with this rotomer are dimeric (*i*.*e*. AmiR (PDB ID 1QO0), HP1043 (PDB ID 2PLN), PDB ID 2QZJ, PDB ID 2ZAY and PDB ID 3HV2; S2 Fig), with some demonstrating DNA binding and activity *in vivo* without phospho-Asp driven changes influencing their conformational equilibrium states (*e*.*g*. HP1043 (PDB: 2PLN) [38] and FrzS (PDB ID 2I6F) [28]). However, in the case of ChxR this trend is contradicted. The available structure reveals that the conserved ChxR Tyr is not only found facing outward, but also participates in dimer formation (S2 Fig; [37]), reinforcing the notion that an observed outward Tyr rotomer in REC/ALR domains does not always result in a monomeric quarternary state [19]. Interestingly, recent data from Kern and colleagues show that the conserved Tyr/Phe and Ser/Thr pair in the response regulator NtrC operate on different timescales in solution, and specifically that the Tyr/Phe does not participate in the active conformation of this protein [54]. Although we did observe that in RitR Tyr100 was important for DNA-binding and *in vivo* repression of *piu* (Fig 5), its precise role in RitR activation still remains to be determined.

### Contribution of key residues to RitR function

To date it remains enigmatic as to how both atypical REC domains and ALRs are activated in the absence of phosphorylation. Do these proteins exhibit equilibrium between active and inactive states and are driven to activation by an input signal through their Y/T-coupling residues? [5] Or are they instead mainly constitutive and impervious to signaling inputs and resulting downstream structural changes? Recent data has revealed that the activity of several ALRs as well as canonical REC domains can be modulated through ligand or PTM mediated signals other than Asp phosphorylation, which includes Ser/Thr phosphorylation [22,42] and direct binding by small molecule activators [20,55]. The observed conservation of the canonical REC Y/T-coupling residues in ALRs shown here (Figs 1a, S1, and S3) suggests that although the ALR inducer can be variable, after this signal induction takes place in many cases some version of the Y/T-coupling relay and dimerization mechanism may work to modulate ALR activity. At present, it still remains unclear as to why most ALRs have retained these residues and have thus far shown to be functionally important *in vivo* in the few examples available (ref. [28] and these studies).

The unique uncoupled state of the RitR Y/T (Y/D in the case of RitR) pair in our structure presented an opportunity to explore the activation of an ALR in the absence of the inducer. To attempt to force RitR to dimerize in the absence an upstream signal we introduced substitutions within divergent acidic triad residues Lys10 and Asn53 (Fig 5a in cyan), Y/T-coupling residues Tyr100 and Asp81 (Fig 5a in orange), and Gate residues Leu86, Leu90, Val93, Tyr102 and Phe96 (Fig 5a in green). Purified RitR mutant and wild-type proteins (S6 Fig) were then assessed for their oligomeric state via Size Exclusion Chromatography (SEC) and the ability to bind to pre-defined RitR target DNA [23] by Electrophoretic Mobility Shift Assays (EMSAs). Furthermore, to examine the contributions of these individual residues to the RitR mediated repression of *piu* iron transporter expression *in vivo* [23,24], we created β-galactosidase reporter strains in *S*. *pneumoniae* where we could measure Piu promoter (P_*piu*_) activity in response to the same RitR isogenic variants (see Methods section for details).

Results shown in Fig 5b–5e indicate that mutagenesis of the RitR acidic triad residue coordinates Lys10 and Asn53 had no effect on the ability of RitR to bind DNA, form a dimer *in vitro* or modulate P_*piu*_ activity *in vivo*. These data suggest that similar to other functionally-characterized ALRs [25,27,28] and also atypical REC domains that possess the phosphorylatable Asp [9], that at least under these tested conditions RitR does not rely on its acidic triad residue positions to function. Conversely, when Y/T-coupling residues Tyr100 or Asp81 were replaced with Ala we observed DNA binding with as little as 0.22 μM of RitR in EMSA experiments (Fig 5c and 5d). This was significant in that previous to these experiments DNA binding with full-length RitR (RitR_FL_) was unobtainable without first removing the ALR (REC) domain (wild-type sample Fig 5c and 5d and ref. [23]), a phenomenon likely due the restraint of the helix-turn-helix motif by intramolecular ALR-DBD domain contacts in the absence of an inducer [19]. SEC experimentation of the Asp81 mutant revealed a split peak profile, neither of which perfectly aligned with the expected monomer or dimer molecular weights, whereas the Tyr100 mutant was mostly monomeric (Fig 5b). However, *in vivo* results show that Asp81 and Tyr100 are required for full repression of *piu* transcription (Fig 5e). As REC/ALR dimers are normally the ‘activated’ form of the protein that binds DNA, the SEC results are apparently in conflict with the EMSA and *in vivo* data. A likely explanation is that in the absence of DNA mutations within Asp81 and Tyr100 destabilize RitR such that it is unable to form a proper dimer *in vitro*. Experimental support for this hypothesis includes our SEC analysis of Y/T-coupling residues Asp81 and Tyr100 in both RitR_FL_ and RitR_ALR_ constructs, as well as our accompanying RitR_ALR_ HSQC spectra, where mutations within these residues produces protein with enhanced aggregation and heterogeneic properties (S4 Fig).

In contrast to mutagenesis of the RitR Y/T-coupling residues, substitutions within Gate residues Leu86 and Leu90 yielded well-behaved protein that resulted in DNA binding, clear dimer formation *in vitro* and an enhancement effect on P_*piu*_ activity *in vivo* (Fig 5). An Ala substitution of Gate residue Val93 alone did not influence RitR DNA binding, but was sufficient to produce more dimer in solution (Fig 5b). However, when the Val93 mutant was combined with a Leu86 mutation, we then observed a large shift in equilibrium towards the dimeric form of RitR_FL_ when compared to the SEC profile of either of these Gate mutations alone (Fig 5b). The Leu86/Val93 double mutant also exhibited DNA binding and modified P_*piu*_ activity *in vivo* (Fig 5e). Mutation of Gate residue Phe96 produced a marked loss of promoter repression *in vivo*, however *in vitro* this mutant displayed a heterogeneous SEC profile and no observed DNA binding activity, suggesting that this residue might be crucial to protein stability. On the other hand a substitution at the Gate residue Tyr102 did not have an effect on RitR DNA binding, modulation of P_*piu*_ activity *in vivo* or result in a clear dimeric state in solution (Fig 5).

From these experiments we can conclude that substitutions within key Gate residues Leu86, Leu90, and Val93 results in the enhancement of dimerization and DNA-binding *in vitro*, and *in vivo* these same mutations cause a derepression and/or activation of P_*piu*_ (Fig 5e). However, these *in vitro* and *in vivo* data are seemingly in conflict, as RitR is classically known as a repressor of *piu* transcription [23,24,56], and thus enhanced DNA binding would be expected to result in maintained P_*piu*_ repression *in vivo*, and not the observed increase seen in P_*piu*_ activity (Fig 5e). In response, recent data from our lab has shown that RitR regulation is much more complex than this simple paradigm, where other PTMs play a pivotal role in its regulatory effects, including Ser-Thr phosphorylation and oxidation ([24] and Wright *et al*. under review). Moreover, we have found that RitR is also able to act as an activator of *piu* transcription when occupying different P_*piu*_ transcriptional regulatory sites, which here we have shown can be artificially induced through Y/T-coupling and Gate mutations (Fig 5e). Future studies will have to determine how PTMs, possibly other transcription factors and changing environmental conditions precisely contribute to the complexity of RitR promoter occupancy and transcriptional control *in vivo*.

### RitR dimerization

**Fig 6:**
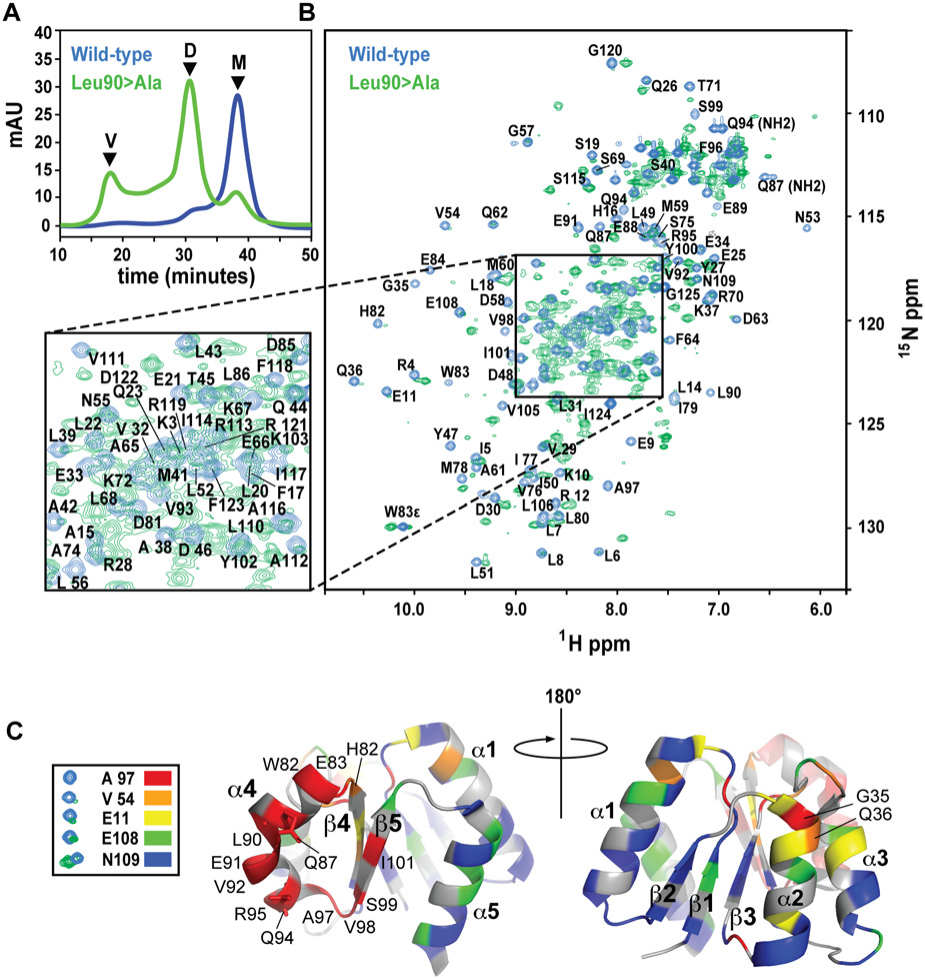
SEC and NMR analysis of wild-type RitR_ALR_ and the RitR_ALR_ Leu90>Ala mutant proteins. (**a**) SEC of the proteins. V, void volume; D, dimer peak; M, monomer peak. mAU, milli Absorbance Units. (**b**) ^1^H-^15^N HSQC spectra of the wild-type RitR_ALR_ (blue peaks) and Leu90>Ala mutant (green peaks). Assigned amides and NH_2_ groups from Gln87 and Gln94 are also labeled. ppm, parts per million. (**c**) Heat map of chemical shift changes between the Leu90>Ala mutant and wild-type proteins. Examples of typical peaks used to calculate the degree of change between the mutant and wild-type for the heat map, and their associated coloring scheme are shown on the left. The RitR heat map structures show the proposed α4-β5 dimeric interface from two perspectives that differ by 180°. Secondary structures and residues that experienced large changes in their chemical environments when transitioning from the wild-type monomer to the mutant dimeric structure are labeled.

Although we observed clear RitR dimerization with substitutions in Gate residues, we wanted to know if the RitR dimer interface was facilitated through the typical α4-β5-α5 face used by regular REC domains and other known ALRs ([27,38]; S2 Fig; S3 Table). To answer this question we collected three-dimensional NMR data with [*U*-^15^N,^13^C] RitR. The NMR spectra displayed excellent peak quality and distribution that would be predicted of a monomeric RitR_ALR_, and were assigned to completion. Next we produced purified ^15^N-labeled protein of the Ala substitution mutants (ALR domain only, or RitR_ALR_) of Leu86, Leu90, Asp81 and Tyr100. When SEC was used to analyze their oligomeric states as expected the wild-type was predominantly monomeric (Figs 6a and S4a). Conversely, when Gate residue mutants were similarly examined (Leu86 and Leu90) they were almost entirely dimeric, with the Leu90 mutant showing a small monomeric peak, and the Leu86 mutant displaying more heterogeneity in this region with two peaks (Figs 6a and S4a). Paralleling the data of full-length protein SEC experiments, the Y/T-coupling residue mutants were more poorly behaved than Gate substituted versions. SEC data showed the Tyr100 RitR_ALR_ mutant to be highly unstable, where most of the protein was found within the void volume (S4a Fig). Moreover the Asp81 mutant formed mainly aggregates after initial Ni^2+^ affinity purification and was therefore not examined further. These results suggest that the RitR DNA-binding domain aids in the stability of the protein in the active form, which could be further stabilized by the presence of its target DNA sequence as is often seen with helix-turn-helix containing transcription factors.

NMR data generally reflected what we observed by SEC, where a new set of peaks likely representing the chemically distinct dimeric state of RitR could be detected in the Leu86 and Leu90 mutants (Figs 6b and S4b). Although not identical, the Leu86 and Leu90 mutant spectra exhibited considerable spectral overlap (S4b Fig), which suggests that these two mutants both form similar dimeric structures. Differences between these spectra could be due the chemical shift contributions within the immediate chemical environment of the mutated residues. When compared to the wild-type HSQC plot, some of the amides from the Leu86/Leu90 mutant samples were found to completely disappear, which we deemed to be in very different chemical environments and contributing to the dimer interface, while other shifts remained in similar chemical environments to varying degrees (Figs 6b and S4b).

To better understand what amino acids were changing their immediate chemical environments within the RitR dimer, we superimposed the wild-type HSQC plot over the most homogeneous mutant sample (Leu90; Fig 6b). Residues that experienced the largest differences in the HSQC spectra were then mapped onto the RitR_ALR_ crystal structure using a color-coded chemical shift intensity scheme (Fig 6c). The largest changes in chemical shifts (*i*.*e*. the peaks in question were either severely diminished in intensity or had completely disappeared in the Leu90 sample) were mostly centered around the α4-β5 face of the protein, which participates in the canonical dimerization region in other ALR/REC structures. In addition to the backbone amide chemical shifts from residues Gln87 and Gln94 being completely absent from the Leu86 and Leu90 mutant HSQC spectra, their respective NH_2_ side chain chemical shifts were also missing (Fig 6b). Indeed, the Gln87 and Gln94 side chains, both of which project into the solvent in the ‘inactive’ monomeric crystal structure, are predicted to form a component of the α4-β5 dimer interface according to other dimeric ALR and typical REC available atomic models, and in our dimeric RitR atomic model (see below). Collectively these results show that Gate residues Leu90 and Leu86 stabilize the inactive form of the α4, and suggest that Gln87/Gln94 participate in the RitR dimer. Other regions of the protein experienced little change save the N-termini of the α1 and α2 helices (Fig 6c).

Our results show that similar to other REC and ALR domains, the α4-β5/Gate region of RitR is likely the major component involved in dimerization of the protein. The observed changes in the α1 and α2 could be explained by another minor and unidentified dimer species in slow exchange with the major α4- β5 dimeric species, or possible intra-protein rearrangements resulting from the larger chemical shift changes in the α4-β5/Gate region. Interestingly, in the Leu86 or Leu90 mutants the α5 helix that would normally complete a canonical dimer face exhibited very little changes in chemical environment, suggesting that this helix does not play a major role in RitR dimer formation. Indeed, the degree of the REC/ALR dimeric interface can vary considerably [15,16,37]. In support of this RitR “α4-β5 only” dimer hypothesis, we found one other ALR structure (PDB ID 3HV2; S5a Fig) and two canonical FixJ regulators ([57,58]; S5a Fig), which all use a α4-β5 dimer interface without α5 participation. Furthermore, when we generated an atomic model of the active RitR dimer based on the structure of the FixJ homolog DctD [57,58] we observed that the amino acids which exhibited the greatest changes in chemical shift/intensities aligned at the α4-β5 dimer interface and were predicted to use the expected crucial Gate residues Leu86, Leu90 and Val93 help form the outward facing inter-protomor contacts (S5b Fig). These contacts also included the two Gln residues (Gln87 and Gln94) whose chemical shifts completely disappeared from their wild-type positions in our Leu86 and Leu90 mutant RitR constructs (Figs 6b, S4b, and S5b). Future three-dimensional structures and further biochemical and genetic analyses will be required to determine the precise contribution of individual residues to dimer formation.

Taken together our structural, biochemical and genetic experiments shown here suggest that similar to other REC and ALR domains, RitR uses its conserved Y/T-coupling residues in concert with its Hydrophobic Gate to initiate and form an active dimer (Figs 6 and S5). However, although RitR likely dimerizes at the same face as typical REC domains, we observe that similar to the ALR 3HV2 and FixJ-type transcriptions factors it uses only the α4- β5 to facilitate this interaction. We found that Gate residues Leu86 and Leu90 were especially important in activation of the dimeric form of RitR, and likely involves a repositioning of the conserved Tyr residue from the outward to inward rotomer. Previous studies of typical REC domains have noted the conservation of hydrophobic residues within the α4-β5 Gate, most notably at the RitR Leu90 and Val93 positions, with Leu86 being more variable (see S1 and S2b Figs). In fact in one study it was found that 261 of 269 surveyed OmpR/PhoB-type response regulators possessed a hydrophobic residue at the RitR Leu90 coordinate [53]. In ALRs this residue is also highly conserved as a hydrophobic amino acid (S1 Fig). A survey of the available ALR atomic structures reveals that Leu90 and Val93 are hydrophobic residues in 7/10 and 9/10 of the sequences, respectively (S2b Fig). Moreover, we found that in ALR structures where dimers were available (ChxR (PDB ID 3Q7R), HP1043 (PDB ID 2PLN), AmiA (PDB ID 1QO0), PDB ID 2QZJ and PBD ID 2ZAY), the equivalent coordinates of Leu86, Leu90 and Val93 in all cases participated in their respective dimer interfaces, including in our RitR activated dimer model structure based on DctD (S5b Fig). Combined with our computational data showing their conservation (Figs 1 and S3), these observations further suggest that many ALRs use some form of Y/T-coupling to modulate their activity, and that Gate residues might have a more general function in both canonical REC and ALR domains to ‘fine tune’ the rotomeric states of the conserved Tyr trigger. Additional support comes through studies from Alber and colleagues [28]. They show that similar to most other ALR structures, the ALR FrzS has an inward or “activated” Tyr rotomer. When mutated little difference was observed in protein structure, however *in vivo* data demonstrated a similar phenotype to a full *frzS* deletion and suggests that FrzS might use a ‘reverse’ version of Y/T coupling [28]. Considering that most ALR structures solved thus far show their Tyr trigger in the inward active state (S2 Fig), this mechanism could be more widespread within the ALR domain family. At present we can only speculate that ALRs use a version of the Y/T-coupling mechanism of activation, which is for now based on the conservation of Gate and Y/T coupling residues, and data from only two existing examples (*i*.*e*. RitR and FtzS). Now that the entire available ALR dataset has been identified (S5 Table), we will be able to more comprehensively study the ALR family of signaling domains to aid in our understanding of their diverse mechanistic roles they play in nature, and importantly how they function in the absence of histidine phosphorylation.

## Methods

### Bioinformatics of ALR sequences

#### ALR sequence retrieval

To identify the complete set of ALR sequences we first downloaded all deposited REC domains (accession number: PF00072.full) as an updated Pfam 26.0 [59] dataset alignment file from the Stockholm database (103,233 total REC sequences). Non-redundant REC sequences were then obtained by employing a Python 2.7 script [60] to yield a subset of 74,816 total unique, non-redundant proteins. In the case of our data presented in Figs 1 and S3 and S1 and S2 Tables that include taxonomic cross-referencing, we obtained a 94,229 total REC sequences subset, as some sequences in Pfam do not have their taxon IDs listed in the Pfam taxonomy table. The missing species that were not represented in the Pfam database include: 81972 (*Arabidopsis lyrata* subsp. Lyrata), 77009 (*Hordeum vulgare* subsp. Spontaneum, or wild barley), 145481 (*Physcomitrella patens* subsp. petraea), 59691 (*Arabidopsis lyrata* subsp. petraea), 112509 (*Hordeum vulgare* subsp. vulgare, or domesticated barley), 86192 (*Pseudomonas chlororaphis* subsp. Aurantiaca), 344680 (*Brassica rapa var*. *perviridis*), 3068 (*Volvox carteri f*. *nagariensis*), 51351 (*Brassica rapa* subsp. Pekinensis), 307796 (*Saccharomyces cerevisiae* YJM789), 9615 (*Canis lupus familiaris*, or dog), 223297 (*Streptomyces refuineus* subsp. Thermotolerans), 31964 (*Clavibacter michiganensis* subsp. Sepedonicus), 213301 (*Coffea liberica* var. dewevrei), 46245 (*Drosophila pseudoobscura pseudoobscura*, or fly), 3658 (*Cucumis melo var*. *cantalupensis*, or fly), 40933 (*Bartonella vinsonii* subsp. Berkhoffi), 366649 (*Xanthomonas fuscans* subsp. Fuscans), 36774 (*Brassica oleracea var*. *italica*, or sprouting broccoli), 66854 (*Saccharothrix mutabilis* subsp. Capreolus), 333 (*Pseudomonas chlororaphis* subsp. Chlororaphis), 147469 (*Staphylococcus sciuri* subsp. Rodentium), 114939 (*Prunus domestica* subsp. Insititia), 137 (*Spirochaetaceae*, *Borrelia burgdorfe* species). These missing ALRs are included in the aforementioned 103,233 REC total.

#### ALR sequence identification

In order to filter all ALR sequences from the PF00072.full alignment, the RitR protein accession was used as a reference. Employing a UniProt gene name search [61], the RitR gene name was cross-referenced to the RitR protein accession number B5E7B7. Thereafter, a generic custom script using Python 2.7 [60] and the biopython.org 1.58 libraries was created (match_sequences.py) in order to filter out aligned proteins of interest. Using position Asn53 for the protein B5E7B7 within PF0072.full, all sequences lacking an Asp at the corresponding Asn53 residue within the alignment were extracted to a dedicated ALR alignment file. Subsequent taxonomic and gene function analyses used this ALR alignment file as a foundation for generating data.

#### ALR lineage and gene function

To calculate taxonomy statistics the Pfam (26.0) FTP archive was used to download individual tables, which enabled the creation of custom table subsets. The custom tables included ones used to store alignment subsets, taxonomy extensions, and the NCBI complete microbial genome database http://www.ncbi.nlm.nih.gov/genomes/lproks.cgi (lproks_1.txt; 2011). Database tables were used to store ALR alignment files for future ALR-specific taxon lineage and gene function calculations. The taxonomy extension table provided a foundation for a simplified search and sort of the protein and strain information by taxon rank. The Completed Genome (CG) table derived from information in the NCBI microbial genome database provided a foundation for calculating protein and strain counts by whole genome, and was representative of the prokaryotic genome information at the time Pfam 26.0 sampled the UniProt [61] protein archive for analysis. Custom Python scripts and advanced database queries provided the execution logic to extract and aggregate the data used in Figs 1 and S1 and S1, S2 and S6 Tables. Effector domain statistics in Fig 1d were generated by coupling the total REC, and separately the ALR protein sequence subset to their corresponding protein architectures, and finally output as a series of Pfam accession numbers. These accessions were then tied to function using ref. [18] as a template.

#### ALR residue contribution

Residue statistics presented in Figs 1a and S3 were calculated using the ALR alignment file, the B5E7B7 accession for RitR, the corresponding ‘invariant’ Glu9, Lys10, Asn53, Asp81 and Tyr100 offsets of interest, and a custom script which calculated total amino acid counts at the corresponding position across all aligned ALR sequences.

### Strains and growth conditions

Cultures of pneumococcus were grown overnight from frozen (-80°C) stocks in CAT medium (0.5% (w/v) tryptone, 1% (w/v) casein digest, 0.1% (w/v) yeast extract, and 5 mg/L choline) containing 0.02% (w/v) glucose and the appropriate antibiotics at 37°C in a humidified 5% CO_2_ incubator. The following day cultures were then diluted 1:10 in Todd Hewitt broth medium (Becton Dickinson) supplemented with 1% (w/v) yeast extract (THY) containing appropriate antibiotics, and the cell density measured periodically at 600 nm using a Biomate 3 spectrophotometer (Thermo Scientific, Waltham, MA).

DNA was transformed using the Competence Stimulating Peptide CSP-1, a generous gift from Donald Morrison, University of Illinois-Chicago. For transformations, *S*. *pneumoniae* cells were inoculated from frozen stocks into THY broth containing the appropriate selection antibiotics and cultured to early exponential phase (*i*.*e*. an *A*_590_ of 0.01–0.03). At this time 100 ng of CSP-1 and 100–200 ng of DNA were then added to a 0.5 ml volume of the culture. The transformation reactions were then placed back into the CO_2_ chamber and incubated for at least another 2 hours before being plated onto Tryptic Soy Blood Agar (TSBA) plates (Becton Dickinson, Oxford, UK) containing 4% (v/v) defibrinated sheep blood (Rockland Immunochemicals, Gilbertsville, PA) with appropriate antibiotics and grown at 37°C under 5% CO_2_ until resistant colonies appeared. The kanamycin (Kan) cassette to delete *ritR* was selected with 200–300 μg/ml Kan, and the erythromycin (Erm) cassette to complement with *ritR* variants was selected with 1 μg/ml Erm. Strains of *E*. *coli* were cultured for plasmid purification overnight with aeration in a 37°C incubator in standard lysogeny broth (LB) medium supplemented with appropriate antibiotics: 50 μg/ml spectinomycin, 34 μg/ml chloramphenicol (Cam), or 50 μg/ml ampicillin (Amp) or the more stable Amp substitute carbenicillin (Carb).

### Construction of strains and plasmids

#### Construction of *ritR* chromosomal deletion and *ritR* variant replacement strains

To construct the *ritR* deletion a Kan resistance cassette, promoter and Rho terminator based on the Janus construct [62] were custom synthesized by GenScript USA Inc. (Piscataway, NJ) and cloned into plasmid pCC1 (Epicentre, Madison, WI) with 5’ and 3’ *ritR* flanking R6 strain DNA sequences. To create the *ΔritR* strain, the synthetic constructs were excised from pCC1 plasmid DNA with *EcoR*I and *BamH*I restriction enzymes, gel purified and transformed into *S*. *pneumoniae* R800 cells as described above. Transformants were selected on TSBA supplemented with 200–300 μg/ml of Kan. Inserts were verified with primers *RitR Kan check F1* and *RitR Kan check R1* for the 5’ site of *ritR*, and *RitR Kan check F2* and *RitR Kan check R2* for the 3’ site of *ritR* (S4 Table).

RitR chromosomal mutant replacements were constructed by transforming another custom synthesized cassette into the *ΔritR* strain (GenScript USA Inc., Piscataway, NJ). The cassette, contained in *E*. *coli* vector pJET1.2 (Thermo Scientific), consists of the *ritR* Open Reading Frame (ORF) followed by a Rho terminator and Erm resistance determinant (*erm* ^*r*^) borrowed from the Cheshire construct [63]. The replacement cassette’s *ritR-erm* ^*r*^ sequence is surrounded by *S*. *pneumoniae* strain R6 DNA homologous to the 5’ and 3’ flanking regions of the *ritR* ORF. This construct enabled us to replace *ritR* in the chromosome and easily counter-select with Erm resistance. RitR mutations were then introduced using a variation on the QuickChange method (Stratagene, La Jolla, CA). Mutagenesis was accomplished by combining forward and reverse primers (20 pmols each) containing the codon change of interest and subsequently performing a PCR reaction with 4 μM of 10 mM dNTPs, 1 μl of 50 mM MgSO_4_, 50 ng of plasmid template DNA and 0.25 units of Platinum *pfx* DNA polymerase (Invitrogen, Carlsbad, CA). The PCR reactions were denatured at 95°C for 1 minute, annealed for 1 minute at 2°C below the calculated annealing temperature, and extended at 68°C for 20 minutes. Reactions were run for 25 cycles before a final extension step at 68°C for 7 minutes, and then cooled to 4°C. The resultant reactions were then purified with a QIAQuick PCR purification column, eluted in 50 μl of water and cut with *Dpn*I restriction enzyme for at least 2 hours at 37°C. 1 μl of the *Dpn*I reaction was then transformed into chemically competent DH5α *E*. *coli* cells followed by antibiotic selection on 50 μg/ml Carb. To screen for mutants resistant colonies were selected and their respective plasmids purified and sequenced at the Genomics Laboratory, MRC Clinical Sciences Centre using an ABI3730xl model sequencer (Applied Biosystems/Life Technologies).

To obtain a linear version of the cassette for transformation into *S*. *pneumoniae*, *E*. *coli* DH5α cells containing the cassette-containing pJET1.2 plasmid were propagated overnight with 50 μg/ml Carb in LB broth, and the plasmid DNA extracted with a GenElute Plasmid Miniprep kit (Sigma-Aldrich, St. Louis, MO) as per the manufacturer’s instructions. The cassette was then amplified by PCR with the Expand Long Template PCR System (Hoffmann-La Roche, Basel, Switzerland) using primers *erm-cassette-F* and *erm-cassette-R* (S4 Table), gel purified and transformed into *S*. *pneumoniae* R800 cells. Pneumococcal transformants were selected on TSBA plates supplemented with 1 μg/ml Erm. Integrants were verified for the correct insertion into the genome by sequencing the 5’ and 3’ junctures using primer pairs 5’-FL-check-F/5’-FL-check-R and 3’-FL-check-F/3’-FL-check-R, respectively (S4 Table), and their inability to grow in 200 μg/ml kanamycin.

#### Construction of LacZ reporter strains

LacZ reporter strains were constructed to monitor *piu* promoter activity using the plasmid PP2 as described by Halfmann *et al*. [64]. The *piu* promoter region was amplified and cloned into PP2 using the melt and reanneal method (see below for details) with primer pairs (*Ppiu*-F1/*Ppiu*(1+2)-R2 and *Ppiu*-F2/*Ppiu*(1+2)-R1) and (*Ppiu*-F1/*Ppiu*(1+2+3)-R2 and *Ppiu*-F2/*Ppiu*(1+2+3)-R1) to generate the two respective products (S4 Table). After melting and reannealing the double-stranded inserts were ligated into *EcoR*I and *BamH*I cut PP2 plasmid, transformed into *E*. *coli* DH5α cells and transformants were selected on LB agar supplemented with 50 μg/ml Carb. Plasmids were sequenced for the correct inserts using primers *PP2-seq-F* and *PP2-seq-R* (S4 Table), and subsequently transformed into *S*. *pneumoniae* R800 cells where the transformants were selected on TSBA supplemented 3 μg/ml tetracycline. PP2 chromosomal integrations were verified by PCR amplification of the 5’ and 3’ crossover junctures using primer combinations *PP2-tet-F*/*PP2-tet-R* and *PP2-bga-F*/PP2-bga-R (S4 Table), respectively, and then sequencing the resultant amplimers.

#### RitR ALR receiver domain (RitR_ALR_) and full-length (RitR_FL_) protein expression strain construction

A His_6_-SUMO fusion expression vector (LifeSensors Inc., Malvern, PA) was used to express large quantities of RitR_ALR_ and RitR_FL_ protein for Size Exclusion Chromatography (SEC), EMSAs, crystallographic and NMR experiments. RitR DNA from *Streptococcus pneumoniae* strain R800 [65] encompassing amino acids Met1-Ile124 (with an extra C-terminal glycine residue) was amplified and cloned into the *Bsa*I site of the vector pE-SUMO to generate the N-terminal His_6_-SUMO fusion to RitR_ALR_ and RitR_FL_ by a restriction-less cloning method [66]. Two separate PCR reactions were run to generate *Bsa*I cohesive ends of RitR_ALR_ using primer pairs (RitR REC F1/RitR REC R2 and RitR REC F2/RitR REC R1) and RitR_FL_ using primer pairs (RitR REC F1/RitR FL R2 and RitR REC F2/RitR FL R1) (S4 Table). The two reactions were then combined and purified with QIAQuick PCR purification columns (Qiagen, Valencia, CA), and eluted in 40 μl of ddH_2_O. Using a thermocycler, the combined insert DNA was melted at 98°C and slowly reannealed to generate the *Bsa*I cohesive ends. Next, the RitR DNA products were phosphorylated using T4 polynucleotide kinase (Promega, Madison, WI) in a reaction supplemented 1 mM ATP at 37°C for 4 hours. The phosphorylated products were then cleaned again using QIAQuick PCR purification columns and then ligated into pE-SUMO cut with *Bsa*I using T4 DNA ligase and LigaFast rapid DNA ligation buffer (Promega, Madison, WI) for 15 minutes at room temperature. Ligation reactions were then transformed into DH5α chemically competent cells and plated on selective media LB medium supplemented with 50 μg/ml Kan. After sequence verification of the correct inserts, to generate the final protein expression strains the resultant plasmids were transformed into *E*. *coli* BL21(DE3), or BL21(DE3)-Star *E*. *coli* cells (Invitrogen, Carlsbad, CA). RitR mutations were constructed using the QuickChange method as described above using the appropriate primers (S4 Table).

#### β-galactosidase assays

To measure pneumococcal β-galactosidase activity LacZ reporter strains were grown to mid-exponential phase at 37°C (OD_600_ 0.4–0.7) under microaerophilic conditions (humidified, 5% CO_2_ chamber). Cells were then placed on ice for 10 minutes, pelleted at 4°C, resuspended in 100 mM sodium phosphate and incubated at 37°C for 10 minutes in the presence of 0.33% Triton X-100 (v/v). Reactions were allowed to run for 15 minutes at 37°C in the presence of 46.1 mM disodium phosphate, 38.4 mM monosodium phosphate, 7.7 mM potassium chloride, 4.56 mM magnesium sulphate, 2 mM β-mercaptoethanol and 4 μM O-nitrophenyl-β-D-galactopyranoside. The reactions were then terminated by the addition of 200 mM sodium carbonate and absorbance measured at OD_420_ using a Biomate 3 spectrophotometer (Thermo Scientific). Miller units were then calculated using the following equation: [(OD_420_)/((OD_600_ of cultured sample) x (volume of sample) x (reaction time))] X 1000.

### RitR Expression and purification

#### Expression and purification of RitR_FL_ for EMSA experiments

To express His_6_-SUMO fusions of RitR_FL_ wild-type and variants for EMSA experiments an initial 10 ml starter culture was grown overnight in LB medium, and then the following day used to inoculate 1L of fresh LB containing 50 μg/ml Kan. Cultures were incubated at 37°C while shaken at 200 rpm until an OD_600_ of approximately 1.0 was reached, at which point 1 mM Isopropyl β-D-1-thiogalactopyranoside (IPTG) was added to induce protein expression and the cells were then further incubated overnight at 20°C. The following day cells were then pelleted at 3,100 x g for 10 minutes and resuspended in His wash buffer (50 mM Tris-HCl pH 8, 150 mM NaCl, 20 mM imidazole). Cells were then lysed with 3 X 90 seconds cycles of sonication followed by an hour incubation at 4°C after the addition of 1% Triton X-100 (v/v). Cell debris was then removed by centrifugation at 12,600 x g for 35 minutes. The resulting supernatants were then applied to 2 mls of Ni^2+^ agarose (Qiagen). The Ni^2+^ beads were washed with either PBS or imidazole wash buffer and then eluted in imidazole elution buffer (50 mM Tris HCl pH 8, 150 mM NaCl, 300 mM imidazole). The pooled His_6_-SUMO-RitR fractions were then treated with 0.1 mg/ml (working concentration) of ULP1 protease (LifeSensors Inc.) and the mixture was subsequently loaded into 6,000–8,000 molecular weight cut-off dialysis tubing (Spectrum Laboratories Inc., Rancho Dominguez, CA) and dialyzed against 4L of protein storage buffer (50 mM TRIS pH 8.0, 150 mM NaCl, 5% glycerol (v/v)) overnight at 4°C. After dialysis, the SUMO protease digests were then passed back over the same Ni^2+^ affinity column and the flow-through sample was then concentrated to approximately 5–10 mg/ml using 15 ml capacity Amicon-10 concentrators (Millipore, Billerica, MA). The resulting concentrated protein was then stored in aliquots at -80°C. S6 Fig shows a Coomassie-stained SDS PAGE gel of the purified mutants and wild-type full-length RitR proteins.

#### Expression and purification of RitR_ALR_ for crystallography

To express RitR_ALR_ protein for crystallization, a single colony of the His_6_-SUMO-RitR_ALR_ expression strain was used to inoculate a 20 ml starter culture overnight, which was, in turn, used to inoculate 2 x 1 L cultures of LB medium the following day. These cultures were then shaken at 37°C and grown to an O.D. of approximately 1.0, at which point the temperature was lowered to 25°C and 0.4 ml of 1.0 M IPTG was added to the flask. The flask was then cultured overnight at 25°C to allow sufficient expression of the recombinant protein. The next day, the cells were harvested by centrifugation at 3,100 x g for 20 min and then resuspended in HisTrap Buffer A (25 mM TRIS pH 8.0, 300 mM NaCl, 10 mM imidazole). The cells were then lysed by sonication (30 second pulses at 60% amplitude with 45-second rest periods for 20 cycles (for a total of 10 minutes of sonication time), followed by the removal of cell debris by centrifugation at 39,000 x g (12,300 rpm) for 1 hour. The approximately 60 mls of clarified lysate were then applied to a 5 ml bed resin HisPur column (Thermo Scientific/Pierce, Rockford, IL) in two runs of 30 ml each and eluted in steps of 5, 15, 50, and 100% Buffer B (25 mM TRIS pH 8.0, 300 mM NaCl, 250 mM imidazole). The fractions containing the His_6_-SUMO-RitR_ALR_ fusion protein were then pooled, 1.0 ml of 80 μM (working concentration) of ULP1 protease (LifeSensors Inc.) was added to remove the N-terminal SUMO tag and the mixture was then loaded into SnakeSkin dialysis tubing (3,500 MWCO; Thermo Scientific/Pierce) and dialysed against 3.5 L of Buffer C (50 mM TRIS pH 8.0, 150 mM NaCl) overnight at 4°C. After dialysis, the SUMO protease digests were then passed back over the same HisPur column to remove the protease and the cleaved His_6_-SUMO tag. After this subtractive purification step, the flow-through fractions were pooled and dialysed against 3.5 L of Buffer D (10 mM Bis-TRIS propane pH 7.5). Finally, the dialysed RitR_ALR_ protein was then concentrated to approximately 5–10 mg/ml for crystallization screening.

#### Expression and purification of RitR_ALR_ variants for NMR analyses

For NMR analysis RitR_ALR_ variants were expressed in *E*. *coli* BL21 DE3 cells in M9 minimal media containing ^15^NH_4_Cl and ^13^C-glucose for three-dimensional experiments, or ^15^NH_4_Cl for two-dimensional experiments and grown at 37°C until an O.D. of 0.8–0.9 was reached. Cultures were then induced with 1 mM IPTG and then incubated overnight (14–16 hours) at 24°C, or alternatively for 6 hours at 30°C. Cells were then pelleted at 5,000 x g for 10 minutes, the supernatant(s) decanted and pellet(s) frozen at -20°C. Frozen cells pellets were then resuspended in 25 mls of 50 mM Tris pH 7.6, 150 mM NaCl, 0.1% (v/v) β-mercaptoethanol (βME), and 100 mM PMSF. The resuspended cells were then sonicated with a Branson cell disruptor using a ¾ inch diameter sonication horn (Branson Ultrasonics Co., Danbury, CT) 4 times with 30-second pulses at 50% amplitude with 1-minute rest periods in between sonications. The resultant cell lysate was then clarified by centrifugation at 11,200 x g for 30 minutes and the supernatant applied to 6,000–8,000 MWCO dialysis tubing and dialyzed against 4 L of Dialysis Buffer 1 (50 mM Tris pH 7.6, 150 mM NaCl, and 1 mM PMSF) for at least 3 hours. After dialysis, the lysate was then applied to a 5 ml Ni-NTA His-Bind resin (Novagen, Madison, WI) gravity column in analytical mode (no mixing of the resin bed) and equilibrated for 20 minutes before washing with 10 bed volumes of Wash Buffer (50 mM Tris-HCl pH 7.6, 150 mM NaCl, 5 mM imidazole). The His_6_-SUMO-RitR_ALR_ protein was then eluted from the column with Elution Buffer (50 mM Tris-HCl pH 7.6, 150 mM NaCl, 300 mM imidazole). The purified His_6_-SUMO-RitR_ALR_ proteins were subsequently dialyzed against 4 L of Dialysis Buffer 2 (50 mM Tris-HCl pH 7.6, 150 mM NaCl). The dialyzed protein was then diluted to 15–20 mls in Dialysis buffer 2 and 40 μl of a working concentration of 150 μM (6 nmoles) of ULP1 protease (LifeSensors Inc.). This SUMO cleavage reaction was then allowed to proceed for 3 hours at room temperature. To eliminate the SUMO-His_6_ tag and liberate RitR_ALR_, the cleaved protein reaction was applied to a Ni-NTA gravity column and the flow through containing the desired protein was collected and concentrated to 0.5–1 mM in Storage Buffer (50 mM Tris-HCl pH 7.6, 450 mM NaCl). Concentrated protein samples were stored at -20°C if not used within 24 hours of purification.

### RitR_ALR_ crystallization and x-ray crystal structure determination

Crystals of RitR_ALR_ were grown by the hanging-drop, vapor diffusion method. Drops were comprised of equal parts protein solution (5–10 mg/ml RitR_ALR_ in 10 mM BIS-TRIS propane, pH 7.5) and crystallization solution (20–25% (w/v) polyethylene glycol (PEG) 3350, 2.5 mM magnesium formate, and 20 mM TRIS, pH 8.5). Long, narrow rods appeared after several days. Crystals were prepared for flash-cooling by sequential soaks in solution containing 30% PEG (w/v) 3350, 5 mM magnesium formate, 20 mM TRIS (pH 8.5), and 5, 10, or 20% glycerol (v/v). Flash-cooling was accomplished by plunging glycerol-soaked crystals into liquid nitrogen. Diffraction data were collected from a 20 x 20 x 200 μm crystal at beam line 21-ID-D of the Life Science Collaborative Access Team at the Advanced Photon Source (APS), Chicago, Illinois. Data were processed with HKL2000 [67]. Initial phases were obtained by molecular replacement using the program PHASER [68] in the CCP4 suite [69] and the structure of the PhoP REC domain (PDB ID 1MVO, ref. [70]) as the starting model. The initial electron density maps were poor. Automatic rebuilding using the AutoBuild program [71] in the PHENIX suite [72] improved the phases considerably, allowing approximately 95% of the polypeptide to be modeled, albeit with several discontinuities. This improved model was then subjected to iterative cycles of maximum likelihood refinement in the program PHENIX.refine [73] and manual model building in COOT [74]. After completing the polypeptide, riding hydrogen atoms were added to the model with REDUCE [75], and ordered solvent molecules were added with PHENIX.refine. At this point, the crystallographic and free R factors converged at 0.160 and 0.195, respectively. Subsequent refinement of anisotropic motion in the form of TLS parameters for groups identified by the TLSMD server [76] resulted in significant reductions in both R factors (R_cryst_ = 0.146, R_free_ = 0.167). After final adjustments to the model, including the addition of glycerol molecules from the cryo-protectant solution, the R factors converged at their final values of R_cryst_ = 0.136 and R_free_ = 0.157. Model quality was assessed using the comprehensive validation tools implemented in the PHENIX suite, including MolProbity [77]. Data collection and refinement statistics are presented in Table 1.

### NMR data acquisition, assignments and analysis

[*U*-^13^C,^15^N] RitR_ALR_ or [*U*-^15^N] RitR variants were resuspended in a buffer containing 40 mM ^2^H-Tris-HCl pH 7.6, 300 mM NaCl, 10% (v/v) D_2_O, and 0.02% (v/v) NaN_3_ and then used to collect ^15^N-edited, ^13^C-aliphatic-edited, and ^13^C-aromatic-edited NOESY spectra on a 600MHz Bruker Advance spectrometer at 25°C. Additional spectra were collected to enable the three-dimensional unambiguous assignments of backbone and side chain resonances of the wild-type sample that included ^1^H-^15^N-HSQC, HNCO, HNCA, HN(CO)CA, HN(CO)CACB, HNCACB, C(CO)NH, H(CCO)NH, HBHA(CO)NH, and HCCH-TOCSY experiments. ^1^H-^15^N-heteronuclear NOE experiments were also collected to determine the relative order or flexibility of protein on the nanosecond-picosecond timescales (for further discussion of many of these experiments see ref. [78]. Backbone resonances were initially assigned by automation using the program GARANT [79] and were confirmed by manual inspection in XEASY [80]. Side chains were assigned using GARANT’s genPeaks command for the HCCH-TOCSY spectrum, and side chain assignments were edited and corrected manually in XEASY. Aromatic protons were assigned manually in XEASY from the ^13^C-aromatic-edited NOESY spectrum.

### SEC experiments

SEC experiments were carried out using an Agilent 1220 Compact HPLC equipped with a 250 x 4.6 mm BioBasic SEC-300A column equilibrated with 50 mM TRIS pH 8.0, 150 mM NaCl and 10 mM DTT. The column was calibrated with the Gel Filtration Molecular Weight Marker kit from Sigma-Aldrich (Cytochrome C (12.4 kDa), Carbonic Anhydrase (29 kDa), Bovine Serum Albumin (66 kDa), and Sweet Potato Amylase (200 kDa)). The wild-type and mutant forms of RitR_FL_ protein were pre-treated with 10 mM DTT, injected (5 μl) onto the column and separated at a flow rate of 0.5 ml/min at ambient temperature.

### Electrophoretic Mobility Shift Assays (EMSAs)

EMSA analysis was conducted using HEX (Hexachlorofluorocein)-labeled double stranded DNA representing the RitR 33-mer Binding Site 2 (BS2) within the Piu promoter [23]. The probes were made by combining at room temperature a forward primer containing the HEX label (BS2-HEX-F) with a complementary and unlabeled reverse primer (BS2–R) (S4 Table). EMSAs were carried out in a final volume of 20 μl containing: 20 mM HEPES pH 7.2, 5 mM MgCl_2_, 1 mM CaCl_2_, 0.1 mM EDTA, 10 mM DTT, 10% (v/v) glycerol, 0.5 μM of the double stranded BS2 or control DNA oligos, 800 ng of Poly(deoxyinosinic-deoxycytidylic) acid (poly dI-dC) and RitR_FL_ protein at concentrations between 0 to 6.6 μM. EMSA reaction mixtures were incubated at room temperature for 10 minutes prior to resolution by 4% non-denaturing 1XTAE (Tris-Acetate EDTA) PAGE. Gels were visualized at 560 nm using a FLA3000 FujiFilm imager. For quantification of RitR_FL_ DNA affinity to BS2 (Fig 4d), a fixed concentration of RitR_FL_ protein (2.2 μM) was used in a reaction with HEX-labeled BS2 as above. EMSAs were done in triplicate and quantified using ImageJ [81].

## Supporting Information

S1 Fig Alignment of selected ALR and canonical REC sequences. The ALR sequences were imported in FASTA format into Clustal X 2.1 [82]. The alignment was then uploaded into MacBoxShade 2.15 (Institute of Animal Health, Pirbright, UK) for visual representation. ALR-carrying pathogenic bacteria are colored in red, Cyanobacteria in blue, Archaea in purple, algae and plants in green, yeast and fungi in yellow, and canonical REC sequences in black. The black boxes are identical residues and the grey boxes are similar residues. The following colored boxes and arrows highlight important residues mentioned in the text: blue, acidic triad residue positions (RitR acidic triad-1: Glu9, acidic triad-2: Lys10, and Asn53); orange, Y/T-coupling residue positions (RitR Asp81 and Tyr100); green, hydrophobic amino acids predicted to help form the α4-β5 dimer interface that include Gate residues Leu86, Leu90, and Val93 (RitR coordinates). The black arrows indicate the conserved Lys-Pro motif. Secondary structure β-sheet and α-helix elements based on the RitR_ALR_ atomic structure are shown above the alignment.
(TIF)

S2 Fig Analysis of the available deposited ALR structures. (**a**) Cartoon representations of the α4- β5-α5 interface (colored green) with Y/T coupling residues (colored orange), the acidic triad and conserved Lys/Pro motif (colored cyan) shown as sticks. Oxygen atoms are colored red and nitrogen atoms blue. Potential electrostatic interactions are shown as black dashed lines. X-1 is acidic triad-1 and X-2 is acidic triad-2 where “X” is any amino acid. The changed ALR phospho-Asp residue is annotated in red. (**b**) Alignment of the ALR domains of the structures in (a). Acidic triad residues are shown with cyan arrows, Y/T-coupling residues with orange arrows, Leu86/Leu90/Val93 equivalent Hydrophobic Gate residues with green arrows, and the conserved “KP” motif with black arrows. Note the conservation with the “KP” motif, Y/T-coupling coordinates, the Hydophobic Gate and acidic triad-1 residues, and conversely the lack of conservation at the acidic triad-2 position. The ALR sequences were imported in FASTA format into Clustal X 2.1 [82]. The alignment was then uploaded into MacBoxShade 2.15 (Institute of Animal Health, Pirbright, UK) for visual representation.
(TIF)

S3 Fig Presence of Y/T-coupling residues in ALRs. Left pie chart: frequency of amino acid substitutions within the conserved Tyr/Phe or Ser/Thr Y/T-coupling residues in all ALRs (taken from Fig 1a). Right pie chart: frequency of amino acid substitutions in the other Y/T-coupling site if one partner is already present. In ALRs if there was no selective pressure for Y/F residues appearing given the presence of an S/T residue (which would indicate independence) then the probability of a Y/F residue appearing given the presence of an S/T residue would be expected to equal the probability of Y/F in the general ALR population (*i*.*e*. 66%). However we observe an enrichment of 11% over the background ALR rate (77%- 66% = 11%). In the converse case, the probability of an S/T appearing given the presence of a Y/F residue has a smaller spread of 5% (66%- 61% = 5%). These observations suggest that there is evolutionary pressure in ALRs for Y/T pairing, although not to the extent observed in canonical REC domains, and that the Y/F residue is more highly retained in ALRs than the S/T.
(TIF)

S4 Fig SEC and two-dimensional NMR analysis of wild-type RitR_ALR_, and RitR_ALR_ Leu90, Leu86 and Tyr100 Ala mutants. (**a**) SEC of wild-type RitR_ALR_, and RitR_ALR_ Leu90, Leu86 and Tyr100 Ala mutants. V, void volume; D, dimer peak; M, monomer peak. mAU, milli Absorbance Units. (**b**) ^1^H-^15^N HSQC overlay of spectra from the wild-type RitR_ALR_ sample (blue peaks), and the Leu90 (green peaks) and Leu86 (orange peaks) mutant samples. ppm, parts per million.
(TIF)

S5 Fig Comparison of ALR and REC structures that use a ‘α4- β5 only’ dimer interface. (**a**) Cartoon/surface representation of the dimer structure of an ALR domain (PDB ID 3HV2 from *Pseudomonas fluorescens* Pf-5) and the REC domain from DctD (PDB ID 1L5Y from *Sinorhizobium meliloti*; [58]). One monomer is depicted in grey and the other in green. Secondary structures are labeled. (**b**) Model of the RitR active dimer with heat map from Fig 6 based on the DctD dimer from (a). The structure was generated using DctD as a molecular template with Swiss Model [84]. Notice that the largest chemical shift changes from RitR map to the same α4-β5 Hydrophobic Gate fold (shown in red) that 3HV2 and DctD structures use to dimerize. Key Gate residues involved in the predicted dimer formation are labeled. The model ‘activated’ structure is shown with both a top view and 90° rotated side view.
(TIF)

S6 Fig Coomassie stained SDS-PAGE gel of full-length RitR mutants. (TIF)

S7 Fig Genome organization of Cys-ALRs that were found adjacent to redox or iron related genes. Enzymes are colored in purple, transporters in orange, signaling/regulatory proteins in blue, RNA binding proteins in green, Cys-ALRs in yellow and hypothetical proteins in grey.
(TIF)

S1 Table Taxonomy statistics of REC sequences versus ALRs. Data were extracted from the Pfam database version 26.0 [59]. The statistics are divided by domain (in black), *i*.*e*. Bacteria, Archaea and Eukaryota. These categories (domains) are further subdivided into that of class, or if no class was available then phylum or domain. A dash mark indicates zero or none. Classes are color-coded according to phylum. From top to bottom the color scheme is as follows: for Bacteria (Prokaryotes): *Proteobacteria*, purple; the class of *Chlamydiae*, hot pink; *Verrucomicrobiae*, salmon; *Firmicutes*, orange; *Bacteriodetes*, green; *Chlorobi*, dark green; *Chloroflexi*, light green; *Cyanobacteria*, cyan; *Acidobacteria*, yellow; *Spirochaetes*, light brown; *Actinobacteria*, blue; and other bacteria whose taxons are represented by a single class or are uncategorized Bacteria, grey; Archaea are colored in pink. The coloring for Eukaryota is as follows: *Bangiophyceae* (red algae), brick red; *Coscinodiscophyceae* (diatoms) and *Pelagophyceae* (heterokont algae), blue; *Chlorophyta* (green algae), forest green; *Streptophyta* and vascular plants, green; *Oligohymenophorea* (ciliate protozoa), orange; fungi, dark grey; yeast, yellow; potential insect/animal sequences and other unclassified Eukaryota REC and ALR domains, light grey. Note: Completed Genome (CG) data are not given for Archaea and Eukaryotic sequences.
(PDF)

S2 Table Output domain statistics of REC sequences versus ALRs. Effector/output domain statistics and their correlation to taxonomy (class) were generated using computational methods described in the Methods section. The domain architecture names are as annotated in the Pfam database. The taxon abbreviations are as follows: Ver, *Verrucomicrobia*; DT, *Deinococcus*-*Thermus*; Pla, *Planctomycetes*; CP, *Candidatus Poribacteria*; Fir, *Firmicutes*; Plc, *Placozoa*; Pro, *Proteobacteria*; Cya, *Cyanobacteria*; Chl, *Chlorophyta*; Bct, *Bacteroidetes*; Aqu, *Aquificae*; Asc, *Ascomycota*; Act, *Actinobacteria*; Bac, *Bacillariophyta*; Chr, *Chrysiogenetes*; Str, *Streptophyta*; Aci, *Acidobacteria*; Spi, *Spirochaetes*; Nit, *Nitrospirae*; Clf, *Chloroflexi*; Bas, *Basidiomycota*; Eur, *Euryarchaeota*; Clb, *Chlorobi*; Mic, *Microsporida*; Def, *Deferribacteres*; Bas, *Basidiomycota*; Fus, *Fusobacteria*; Art, *Arthropoda*; Fib, *Fibrobacteres*; Cni, *Cnidaria*; Gem, *Gemmatimonadetes*.
(PDF)

S3 Table Comparison of deposited ALR atomic structures. (PDF)

S4 Table Primers used in this study. (PDF)

S5 Table ALR sequences and accession numbers. (XLS)

S6 Table ALR effector domains and phylogenetic associations. (XLS)
