# Correction: The Aspartate-Less Receiver (ALR) Domains: Distribution, Structure and Function

**DOI:** 10.1371/journal.ppat.1004951

**Published:** 2015-06-18

**Authors:** The PLOS Pathogens Staff

There is an error in Fig 2 that was introduced by the publisher. Specifically, Fig 2 in the published article contains a part C, which was moved to the Supporting Information files during production of the article. Part C is included as Figure S7. The publisher apologizes for this error.

Please see the correct version of Fig 2 here. The figure legend is unchanged.

**Fig 2:**
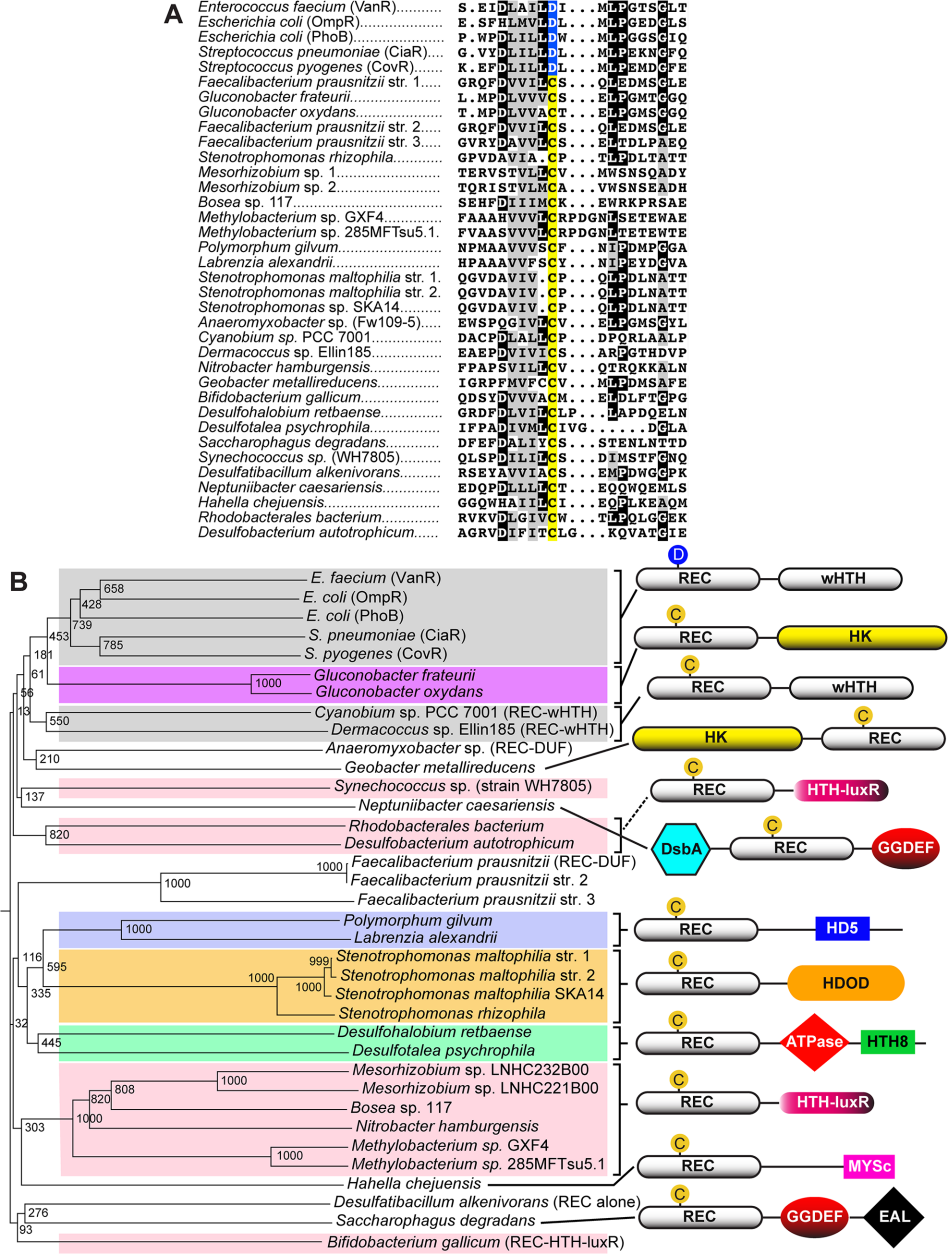
Cys-ALRs. **(a)** Alignment of the 26 extracted ALR domains that contain a cysteine residue in place of the typical phosphorylated Asp seen in canonical REC domain sequences (colored yellow). For comparison, Asp-containing REC domains VanR, OmpR, PhoB, CiaR and CovR were included and their conserved phosphorylatable Asp residue (colored blue). The ALR sequences were imported in FASTA format into Clustal X 2.1 [82]. The alignment was then uploaded into MacBoxShade 2.15 (Institute of Animal Health, Pirbright, UK) for visual representation. **(b)** Phylogenetic tree of Cys-ALRs shown in (a). Related clades are grouped by color and a schematic representation of their domain architecture is shown on the right. Posterior probabilities are shown at the branch points. The circle with a “C” or “D” indicates a Cys or Asp amino acid, respectively, located at the phospho-Asp position. Domain architecture abbreviations are as follows: REC, receiver; HK, histidine kinase; HTH, helix-turn-helix; wHTH, winged helix-turn-helix; DsbA, bacterial disulfide oxidoreductase; GGDEF, cyclic-di-GMP; EAL, diguanylate phosphodiesterase; HDOD and HD5, phosphohydrolase; HTH-luxR, luxR family of bacterial transcription factors; MYSc, myocin domain; DUF, domain of unknown function. The alignment was generated using Clustal X 2.1 [82] and uploaded for phylogenetic display into Archaeopteryx [83].
