# Supplementary figures and images for: The Aspartate-Less Receiver (ALR) Domains: Distribution, Structure and Function
Source: PLoS Pathog. 2015 Apr 13;11(4):e1004795. doi: 10.1371/journal.ppat.1004795 (PMC4395418; doi:10.1371/journal.ppat.1004795)

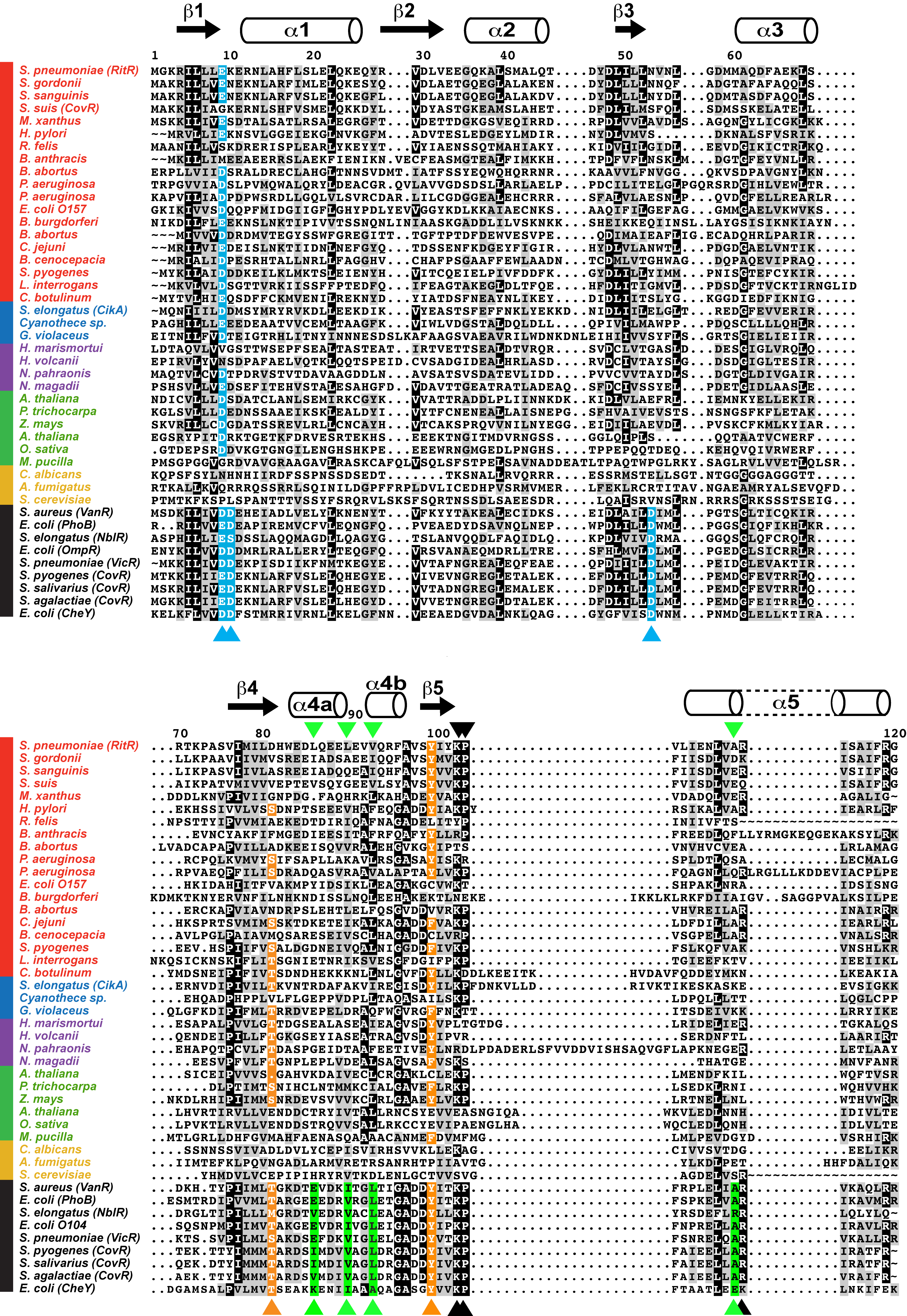

Supplement: S1 Fig — The ALR sequences were imported in FASTA format into Clustal X 2.1 [82]. The alignment was then uploaded into MacBoxShade 2.15 (Institute of Animal Health, Pirbright, UK) for visual representation. ALR-carrying pathogenic bacteria are colored in red, Cyanobacteria in blue, Archaea in purple, algae and plants in green, yeast and fungi in yellow, and canonical REC sequences in black. The black boxes are identical residues and the grey boxes are similar residues. The following colored boxes and arrows highlight important residues mentioned in the text: blue, acidic triad residue positions (RitR acidic triad-1: Glu9, acidic triad-2: Lys10, and Asn53); orange, Y/T-coupling residue positions (RitR Asp81 and Tyr100); green, hydrophobic amino acids predicted to help form the α4-β5 dimer interface that include Gate residues Leu86, Leu90, and Val93 (RitR coordinates). The black arrows indicate the conserved Lys-Pro motif. Secondary structure β-sheet and α-helix elements based on the RitRALR atomic structure are shown above the alignment. (TIF) [file ppat.1004795.s001.tif]

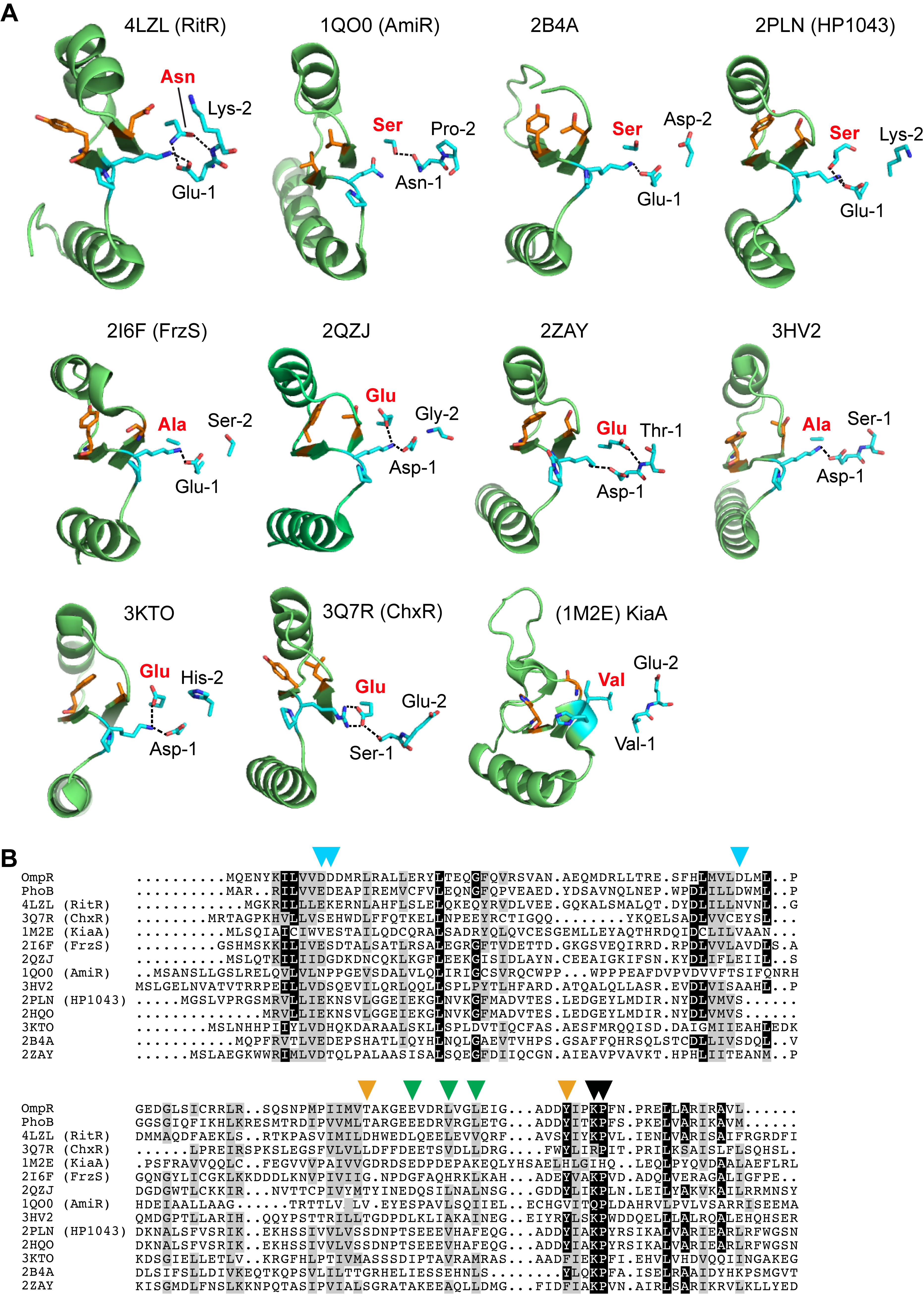

Supplement: S2 Fig — (a) Cartoon representations of the α4- β5-α5 interface (colored green) with Y/T coupling residues (colored orange), the acidic triad and conserved Lys/Pro motif (colored cyan) shown as sticks. Oxygen atoms are colored red and nitrogen atoms blue. Potential electrostatic interactions are shown as black dashed lines. X-1 is acidic triad-1 and X-2 is acidic triad-2 where “X” is any amino acid. The changed ALR phospho-Asp residue is annotated in red. (b) Alignment of the ALR domains of the structures in (a). Acidic triad residues are shown with cyan arrows, Y/T-coupling residues with orange arrows, Leu86/Leu90/Val93 equivalent Hydrophobic Gate residues with green arrows, and the conserved “KP” motif with black arrows. Note the conservation with the “KP” motif, Y/T-coupling coordinates, the Hydophobic Gate and acidic triad-1 residues, and conversely the lack of conservation at the acidic triad-2 position. The ALR sequences were imported in FASTA format into Clustal X 2.1 [82]. The alignment was then uploaded into MacBoxShade 2.15 (Institute of Animal Health, Pirbright, UK) for visual representation. (TIF) [file ppat.1004795.s002.tif]

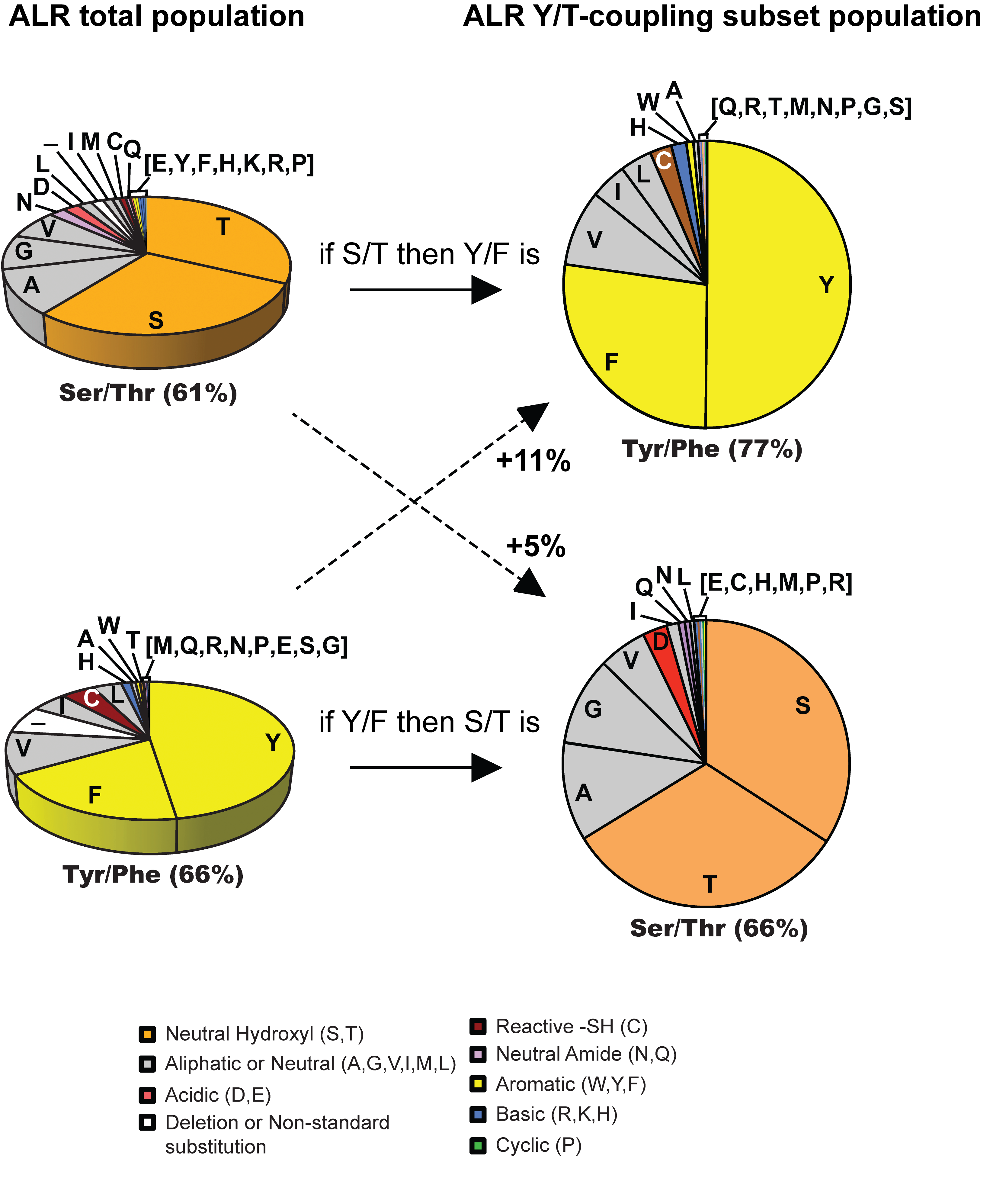

Supplement: S3 Fig — Left pie chart: frequency of amino acid substitutions within the conserved Tyr/Phe or Ser/Thr Y/T-coupling residues in all ALRs (taken from Fig 1a). Right pie chart: frequency of amino acid substitutions in the other Y/T-coupling site if one partner is already present. In ALRs if there was no selective pressure for Y/F residues appearing given the presence of an S/T residue (which would indicate independence) then the probability of a Y/F residue appearing given the presence of an S/T residue would be expected to equal the probability of Y/F in the general ALR population (i.e. 66%). However we observe an enrichment of 11% over the background ALR rate (77%- 66% = 11%). In the converse case, the probability of an S/T appearing given the presence of a Y/F residue has a smaller spread of 5% (66%- 61% = 5%). These observations suggest that there is evolutionary pressure in ALRs for Y/T pairing, although not to the extent observed in canonical REC domains, and that the Y/F residue is more highly retained in ALRs than the S/T. (TIF) [file ppat.1004795.s003.tif]

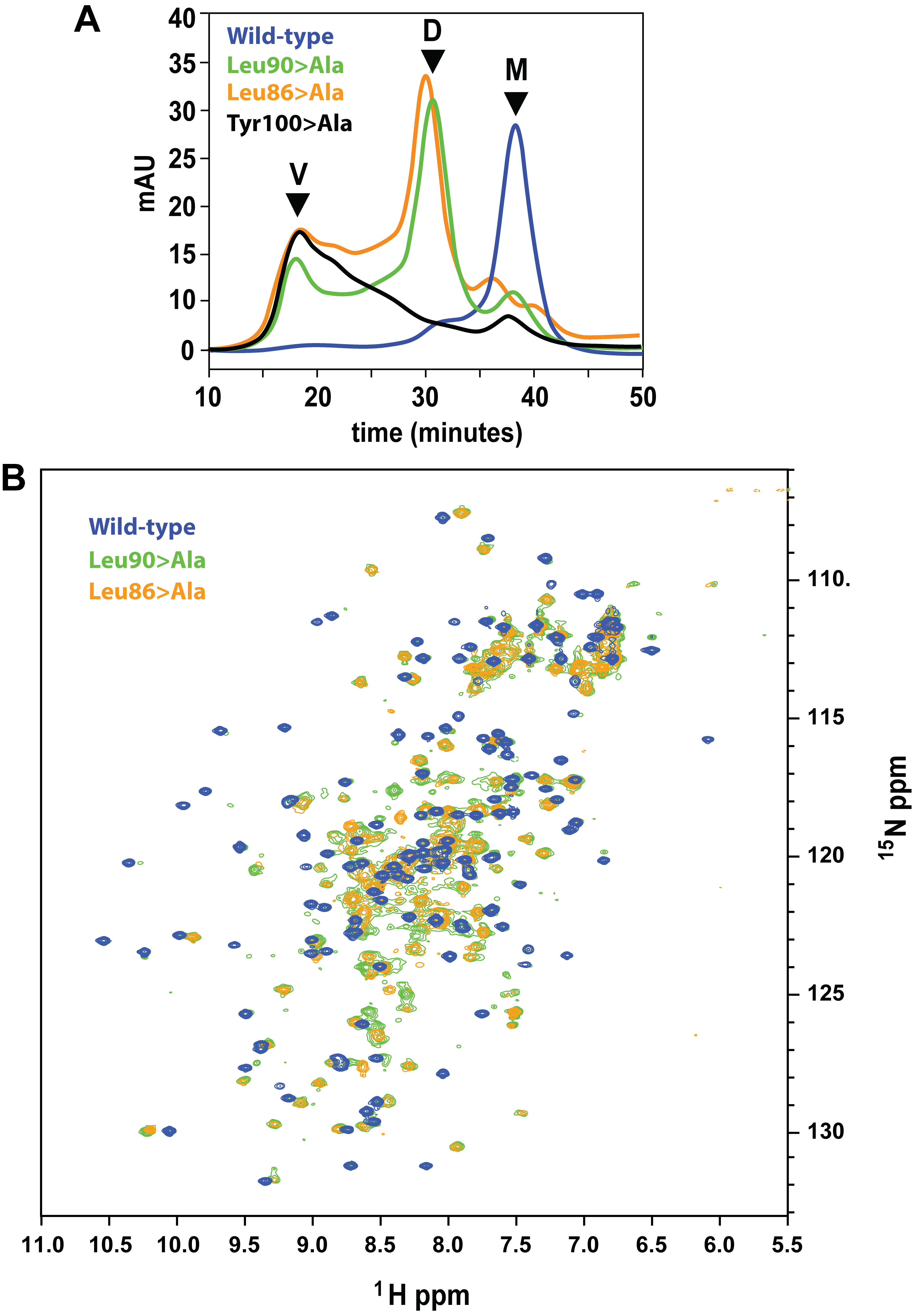

Supplement: S4 Fig — (a) SEC of wild-type RitRALR, and RitRALR Leu90, Leu86 and Tyr100 Ala mutants. V, void volume; D, dimer peak; M, monomer peak. mAU, milli Absorbance Units. (b) 1H-15N HSQC overlay of spectra from the wild-type RitRALR sample (blue peaks), and the Leu90 (green peaks) and Leu86 (orange peaks) mutant samples. ppm, parts per million. (TIF) [file ppat.1004795.s004.tif]

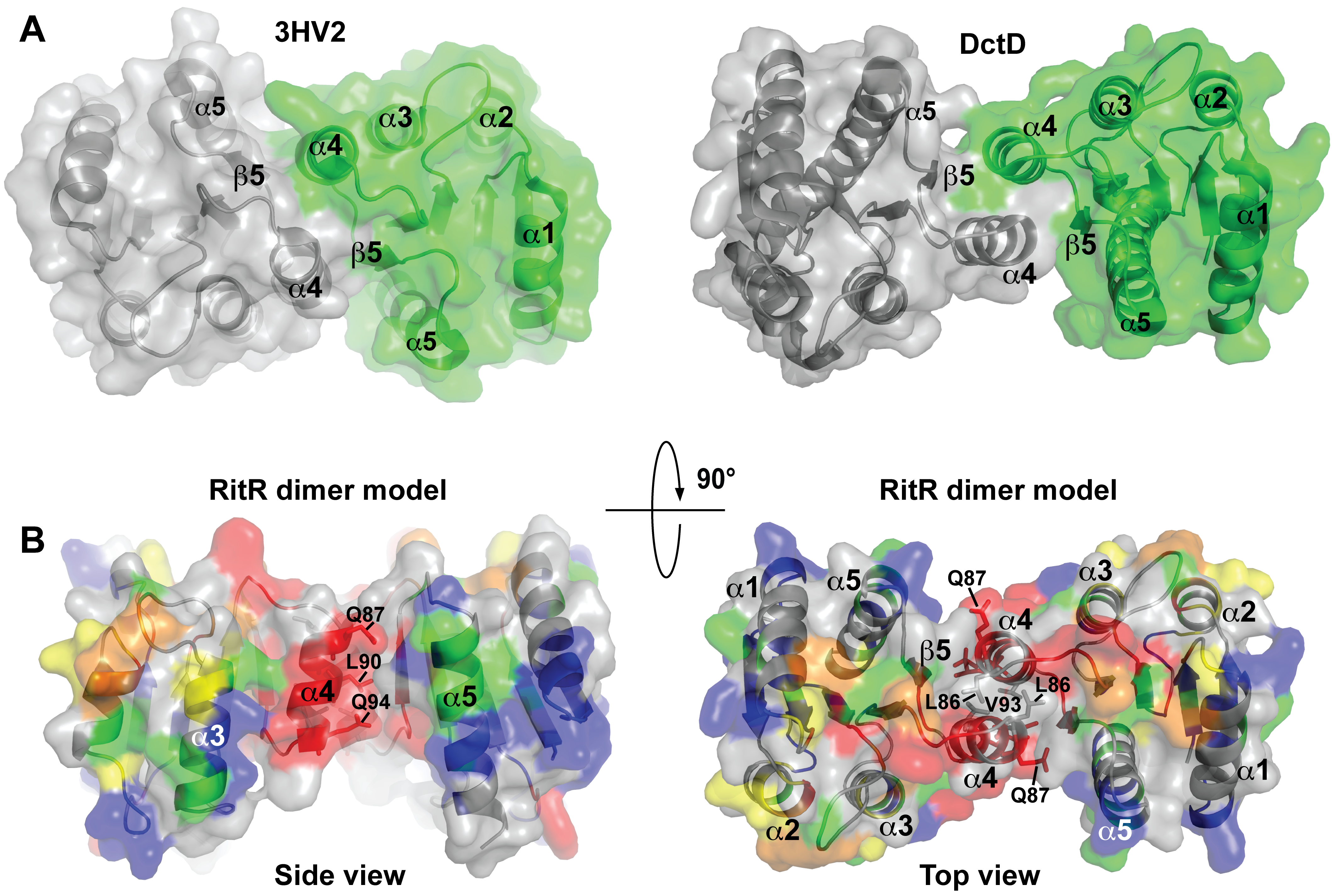

Supplement: S5 Fig — (a) Cartoon/surface representation of the dimer structure of an ALR domain (PDB ID 3HV2 from Pseudomonas fluorescens Pf-5) and the REC domain from DctD (PDB ID 1L5Y from Sinorhizobium meliloti; [58]). One monomer is depicted in grey and the other in green. Secondary structures are labeled. (b) Model of the RitR active dimer with heat map from Fig 6 based on the DctD dimer from (a). The structure was generated using DctD as a molecular template with Swiss Model [84]. Notice that the largest chemical shift changes from RitR map to the same α4-β5 Hydrophobic Gate fold (shown in red) that 3HV2 and DctD structures use to dimerize. Key Gate residues involved in the predicted dimer formation are labeled. The model ‘activated’ structure is shown with both a top view and 90° rotated side view. (TIF) [file ppat.1004795.s005.tif]

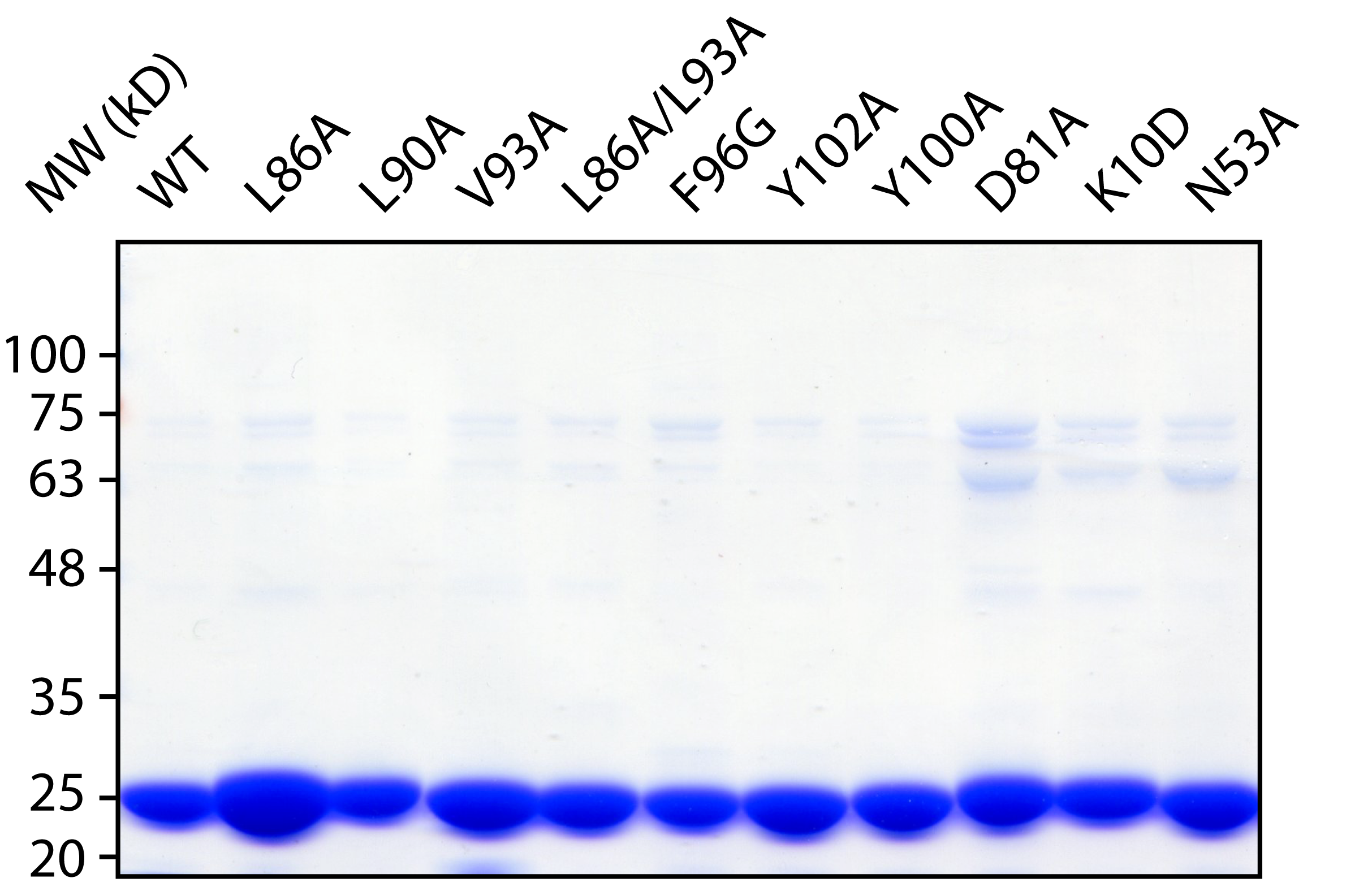

Supplement: S6 Fig — (TIF) [file ppat.1004795.s006.tif]

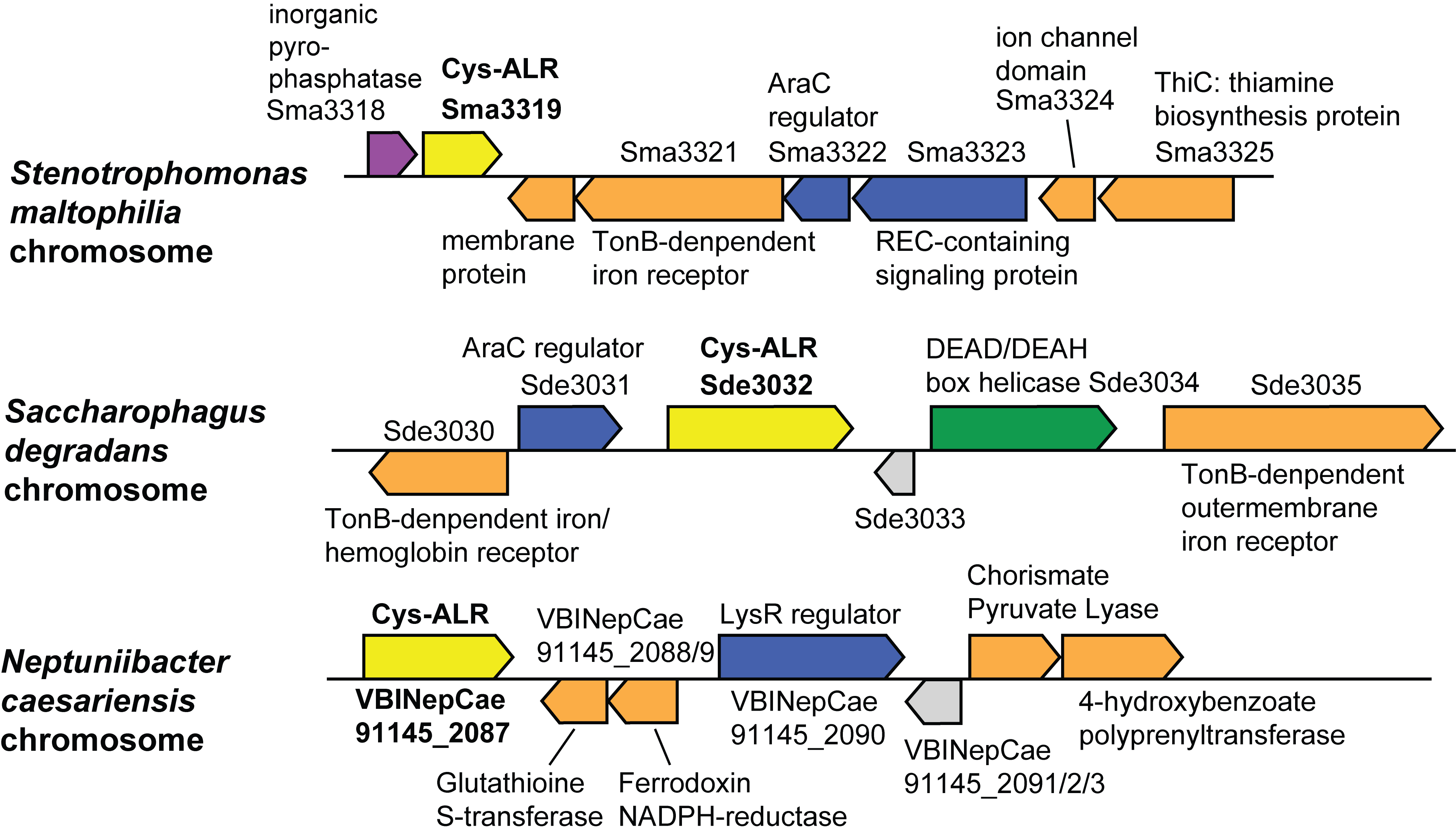

Supplement: S7 Fig — Enzymes are colored in purple, transporters in orange, signaling/regulatory proteins in blue, RNA binding proteins in green, Cys-ALRs in yellow and hypothetical proteins in grey. (TIF) [file ppat.1004795.s007.tif]
