# Supplementary material for: The Aspartate-Less Receiver (ALR) Domains: Distribution, Structure and Function
Source: PLoS Pathog. 2015 Apr 13;11(4):e1004795. doi: 10.1371/journal.ppat.1004795 (PMC4395418; doi:10.1371/journal.ppat.1004795)
Supplement: S1 Table — Data were extracted from the Pfam database version 26.0 [59]. The statistics are divided by domain (in black), i.e. Bacteria, Archaea and Eukaryota. These categories (domains) are further subdivided into that of class, or if no class was available then phylum or domain. A dash mark indicates zero or none. Classes are color-coded according to phylum. From top to bottom the color scheme is as follows: for Bacteria (Prokaryotes): Proteobacteria, purple; the class of Chlamydiae, hot pink; Verrucomicrobiae, salmon; Firmicutes, orange; Bacteriodetes, green; Chlorobi, dark green; Chloroflexi, light green; Cyanobacteria, cyan; Acidobacteria, yellow; Spirochaetes, light brown; Actinobacteria, blue; and other bacteria whose taxons are represented by a single class or are uncategorized Bacteria, grey; Archaea are colored in pink. The coloring for Eukaryota is as follows: Bangiophyceae (red algae), brick red; Coscinodiscophyceae (diatoms) and Pelagophyceae (heterokont algae), blue; Chlorophyta (green algae), forest green; Streptophyta and vascular plants, green; Oligohymenophorea (ciliate protozoa), orange; fungi, dark grey; yeast, yellow; potential insect/animal sequences and other unclassified Eukaryota REC and ALR domains, light grey. Note: Completed Genome (CG) data are not given for Archaea and Eukaryotic sequences. (PDF) [file ppat.1004795.s008.pdf]

Table S1. ALR Taxonomy Statistics[a]

BACTERIA[b]	Rank[c]	RECs[d]	RECs in CGs[e]	CGs[f]	CGs w/ RECs[g]	REC Max[h]	REC Min[i]	Total ALRs[j]	ALRs in CGs[k]	CGs w/ ALRs[l]	ALR Max[m]	ALR Min[n]	Avg. ALRs /CG[o]	Avg. ALRs/ REC CG[p]
Proteobacteria	phylum	176	173	2	1	173	173	6	5	1	5	5	2.50	5.00
Alphaproteobacteria	class	10100	6073	322	129	185	2	504	265	101	8	1	0.82	2.05
Betaproteobacteria	class	8359	4913	187	75	190	3	245	123	50	12	1	0.66	1.64
Deltaproteobacteria	class	6175	4697	64	40	336	9	265	200	39	22	1	3.13	5.00
Epsilonproteobacteria	class	1305	758	73	36	78	9	119	67	34	4	1	0.92	1.86
Gammaproteobacteria	class	23386	10420	746	242	140	1	466	159	96	7	1	0.21	0.66
Zetaproteobacteria	class	67	-	1	-	-	-	1	-	-	-	-	0	0
Chlamydiae (class)	class	37	24	25	19	4	1	1	-	-	-	-	0	0
Lentisphaerae	phylum	39	-	2	-	-	-	1	-	-	-	-	0	0
Verrucomicrobia	phylum	16	12	2	1	12	12	2	1	1	1	1	0.50	1.00
Opitutae	class	233	203	3	2	162	41	12	10	2	9	1	3.33	5.00
Spartobacteria	class	203	-	1	-	-	-	-	-	-	-	-	0	0
Verrucomicrobiae	class	315	5	3	1	5	5	12	-	-	-	-	0	0
Bacilli	class	12060	3634	596	157	144	2	239	77	59	9	1	0.13	0.49
Clostridia	class	7167	2948	218	89	94	1	214	102	52	5	1	0.47	1.15
Erysipelotrichi	class	360	6	16	1	6	6	11	1	1	1	1	0.06	1.00
Negativicutes	class	151	24	18	3	12	3	3	-	-	-	-	0	0
Bacteroidetes	phylum	36	3	3	1	3	3	3	-	-	-	-	0	0
Bacteroidia	class	2959	355	77	12	49	6	38	4	4	1	1	0.05	0.33
Cytophagia	class	590	435	8	5	149	41	15	11	5	4	1	1.38	2.20
Flavobacteria	class	845	454	26	12	87	5	37	16	8	3	1	0.62	1.33
Sphingobacteria	class	755	363	12	5	154	34	21	13	5	4	1	1.08	2.60
Chlorobia	class	221	187	12	10	50	3	12	12	6	6	1	1.00	1.20
Chloroflexi	class	712	595	7	6	125	89	24	19	6	6	1	2.71	3.17
Dehalococcoidetes	class	159	159	6	6	33	19	9	9	6	2	1	1.50	1.50
Ktedonobacteria	class	175	-	1	-	-	-	14	-	-	-	-	0	0
Thermomicrobia	class	55	55	2	2	38	17	4	4	1	4	4	2.00	2.00
Cyanobacteria	phylum	3158	2002	75	39	195	5	202	116	37	9	1	1.55	2.97
Gloeobacteria	class	56	-	1	-	-	-	3	-	-	-	-	0	0
Acidobacteria	phylum	127	120	3	1	120	120	6	6	1	6	6	2.00	6.00
Acidobacteria	class	207	207	4	4	60	45	9	9	4	3	2	2.25	2.25
Solibacteres	class	162	162	1	1	162	162	6	6	1	6	6	6.00	6.00
Fibrobacteres	class	30	60	1	1	60	60	2	4	1	4	4	4.00	4.00

Spirochaetes	class	751	493	47	20	99	3	51	32	17	4	1	0.68	1.60
Actinobacteria	class	7431	3439	392	112	119	1	146	77	47	6	1	0.20	0.69
Aquificae	class	93	63	9	5	24	8	2	-	-	-	-	0	0
Chrysiogenetes	class	83	83	1	1	83	83	7	7	1	7	7	7.00	7.00
Deferribacteres	class	132	132	2	2	73	59	3	3	2	2	1	1.50	1.50
Deinococci	class	310	271	13	11	51	13	6	6	4	2	1	0.46	0.55
Fusobacteria	class	246	59	25	4	31	5	6	1	1	1	1	0.04	0.25
Gemmatimonadetes	class	81	81	1	1	81	81	1	1	1	1	1	1.00	1.00
Nitrospira	class	214	157	7	2	116	41	8	8	2	5	3	1.14	4.00
Planctomycetacia	class	526	221	10	4	64	49	36	15	4	6	3	1.50	3.75
Candidatus Poribacteria	phylum	6	-	1	-	-	-	1	-	-	-	-	0	0
Bacteria	kingdom	197	35	36	1	35	35	12	1	1	1	1	0.03	1.00
**ARCHAEA[b]**	**Rank[c]**	**RECs[d]**	**RECs in CGs[e]**	**CGs[f]**	**CGs w/ RECs[g]**	**REC Max[h]**	**REC Min[i]**	**Total ALRs[j]**	**ALRs in CGs[k]**	**CGs w/ ALRs[l]**	**ALR Max[m]**	**ALR Min[n]**	**Avg. ALRs /CG[o]**	**Avg. ALRs/ REC CG[p]**
Euryarchaeota	phylum	27	22	5	2	14	8	1	1	1	1	1	0.20	0.50
Halobacteria	class	237	231	16	12	41	5	52	51	11	9	1	3.19	4.25
Methanobacteria	class	25	23	3	2	12	11	1	1	1	1	1	0.33	0.50
Methanomicrobia	class	355	351	15	14	89	1	15	15	7	5	1	1.00	1.07
**EUKARYOTA[b]**	**Rank[c]**	**RECs[d]**	**RECs in CGs[e]**	**CGs[f]**	**CGs w/ RECs[g]**	**REC Max[h]**	**REC Min[i]**	**Total ALRs[j]**	**ALRs in CGs[k]**	**CGs w/ ALRs[l]**	**ALR Max[m]**	**ALR Min[n]**	**Avg. ALRs /CG[o]**	**Avg. ALRs/ REC CG[p]**
Bangiophyceae	class	11	-	6	-	-	-	1	-	-	-	-	-	-
Coscinodiscophyceae	class	8	-	1	-	-	-	1	-	-	-	-	-	-
Pelagophyceae	class	6	-	1	-	-	-	1	-	-	-	-	-	-
Cryptophyta	class	5	-	3	-	-	-	1	-	-	-	-	-	-
Chlorophyceae	class	11	-	1	-	-	-	1	-	-	-	-	-	-
Trebouxiophyceae	class	7	-	1	-	-	-	2	-	-	-	-	-	-
Unnamed *Chlorophyta*	class	41	-	4	-	-	-	2	-	-	-	-	-	-
Streptophyta	phylum	510	-	56	-	-	-	88	-	-	-	-	-	-
Coniferopsida	class	5	-	2	-	-	-	1	-	-	-	-	-	-
Liliopsida	class	296	-	14	-	-	-	75	-	-	-	-	-	-
Isoetopsida	class	56	-	1	-	-	-	3	-	-	-	-	-	-
Oligohymenophorea	class	257	-	2	-	-	-	12	-	-	-	-	-	-
Dothideomycetes	class	122	-	9	-	-	-	6	-	-	-	-	-	-
Eurotiomycetes	class	368	-	37	-	-	-	58	-	-	-	-	-	-
Leotiomycetes	class	58	-	4	-	-	-	4	-	-	-	-	-	-
Sordariomycetes	class	168	-	18	-	-	-	14	-	-	-	-	-	-
Pezizomycetes	class	12	-	1	-	-	-	1	-	-	-	-	-	-
Agaricomycetes	class	54	-	7	-	-	-	6	-	-	-	-	-	-

Microsporidia	phylum	5	-	2	-	-	-	2	-	-	-	-	0	0
Saccharomycetes	class	140	-	30	-	-	-	21	-	-	-	-	0	0
Schizosaccharomycetes	class	11	-	2	-	-	-	1	-	-	-	-	0	0
Arachnida	class	3	-	1	-	-	-	1	-	-	-	-	0	0
Anthozoa	class	17	-	1	-	-	-	1	-	-	-	-	0	0
Placozoa	phylum	4	-	1	-	-	-	1	-	-	-	-	0	0
Eukaryota	kingdom	107	-	13	-	-	-	11	-	-	-	-	0	0
unclassified sequences	kingdom	155	-	8	-	-	-	8	-	-	-	-	0	0

[a]Statistics derived from receiver domains (PF00072) obtained from the Pfam database version 26.0; REC, two-component response regulator receiver domain; ALR, Aspartate-Less Receiver domain; CG, Complete Genomes; Statistics are colored in accordance with Phyla; Bacterial Phyla with a single Class are colored in grey, with the exception of the *Spirochaetes*, *Actinobacteria, Chlorobia, Fibrobacteres and Chlamydiae.*

[b]Kingdom

[c]Class rank- higher ranks are given if a Class does not exist (*i.e.* phylum, and kingdom).

[d]Total number of REC sequences within the given rank, both in complete and incomplete genomes.

[e]Total number of REC domains within complete genomes only.

[f]Total number of complete genomes in the given rank.

[g]Total number of complete genomes that contain at least one REC sequence.

[h]Maximum number of REC sequences within a single complete genome in the given rank.

[i]Minimum number of REC sequences within a single complete genome in the given rank.

[j]Total number of ALR sequences within the given rank, both in complete and incomplete genomes.

[k]Total number of ALR domains only within complete genomes.

[l]Total number of complete genomes that contain at least one ALR sequence.

[m]Maximum number of ALR sequences within a complete genome in the given rank.

[n]Minimum number of ALR sequences within a complete genome in the given rank.

[o]The average number of ALRs per total number of complete genomes within a given rank.

[p]The average number of ALRs per number of complete genomes that contain at least one REC domain within a given rank.
