# Supplementary material for: The Aspartate-Less Receiver (ALR) Domains: Distribution, Structure and Function
Source: PLoS Pathog. 2015 Apr 13;11(4):e1004795. doi: 10.1371/journal.ppat.1004795 (PMC4395418; doi:10.1371/journal.ppat.1004795)
Supplement: S2 Table — Effector/output domain statistics and their correlation to taxonomy (class) were generated using computational methods described in the Methods section. The domain architecture names are as annotated in the Pfam database. The taxon abbreviations are as follows: Ver, Verrucomicrobia; DT, Deinococcus-Thermus; Pla, Planctomycetes; CP, Candidatus Poribacteria; Fir, Firmicutes; Plc, Placozoa; Pro, Proteobacteria; Cya, Cyanobacteria; Chl, Chlorophyta; Bct, Bacteroidetes; Aqu, Aquificae; Asc, Ascomycota; Act, Actinobacteria; Bac, Bacillariophyta; Chr, Chrysiogenetes; Str, Streptophyta; Aci, Acidobacteria; Spi, Spirochaetes; Nit, Nitrospirae; Clf, Chloroflexi; Bas, Basidiomycota; Eur, Euryarchaeota; Clb, Chlorobi; Mic, Microsporida; Def, Deferribacteres; Bas, Basidiomycota; Fus, Fusobacteria; Art, Arthropoda; Fib, Fibrobacteres; Cni, Cnidaria; Gem, Gemmatimonadetes. (PDF) [file ppat.1004795.s009.pdf]

Table S2. ALR Effector Domain (ED) Statistics[a]

DOMAIN ARCHITECTURE[b]	FAMILY NAME[c]	PFAM ENTRY[d]	FUNCTION[e]	PDB ID[f]	TAXON (CLASS)[g]	ALR (#)[h]	ALR (%)[i]	REC (#)[j]	REC (%)[k]	ALR/ REC (%)[l]
REC	REC-alone	PF00072	various	1ehc	Ver, DT, Pla, CP, Fir, Plc, Pro, Cya, Chl, Bct, Aqu, Asc, Act, Bac, Chr, Str, Aci, Spi, Nit, Clf, Bas, Eur	613	17.60	14516	13.74	4.2
REC-Trans_reg_C	OmpR/PhoB	PF00486	DNA-binding	1kgs	Ver, Clb, Fir Pro, Cya, Bct, Act, Aci, Str, Spi, Clf	609	17.48	30299	14.48	2.0
Various	other	various	various	na		587	16.85	18519	18.48	3.1
REC-GerE	LuxR/GerE	PF00196	DNA-binding	1a04	Ver, Pla, Fir, Pro, Cya, Bct, Act, Aci, Spi, Nit, Clf, Mic	394	11.31	13771	18.08	2.8
REC-REC-GGDEF	GGDEF	PF00990	Diguanylate cyclase	2v0n	Pro, Cya, Def	165	4.74	326	0.32	50.6
REC-CCT	CCT	PF06203	Plant circadian rhythm	na	Chl, Str	163	4.68	179	0.18	91.0
REC-REC	REC	PF00072	Unknown	1ehc	Ver, Clb, Pla, Fir, Pro, Asc, Spi, Bas	131	3.76	244	0.24	53.6
REC-LytTR	LytR/AgrA	PF04397	DNA-binding	3bs1	Fir, Pro, Bct, Act, Aci	117	3.36	4007	2.42	2.9
REC-ANTAR	AmiR	PF03861	RNA binding	1qo0	Pla, Fir, Pro, Act, Spi	92	2.64	822	0.74	11.1
REC-Sigma54_activat-HTH8	Sigma54-Fis	PF00158 PF02954	Transcriptional control	1ojl; 1etk	Ver, Pla, Fir, Fus, Pro, Bct, Str, Aci, Spi, Art, Fib	88	2.53	5479	4.00	1.6
Pkinase-Pkinase-REC	Ser-Thr kinase	PF00069	Protein kinases (Ser, Thr, Tyr)	1jwh	Asc	69	1.98	79	0.08	87.3
REC-GGDEF	GGDEF	PF00990	Diguanylate cyclase	2v0n	Fir, Pro, Cya, Act	63	1.81	742	0.65	8.4
REC CheB_methylest	CheB	PF01339	Glutamate methyltransferases	1a2o	Fir, Pro, Cya, Spi	44	1.26	1672	1.17	2.6
REC-AAA31	AAA31	PF13614	ATPases	2ved	Pla, Fir, Pro, Act, Clf	44	1.26	187	0.18	23.5
HisKA-HATPase_c-REC	Histidine kinase	PF02518 PF00512	Histidine kinases	2c2a	Fir, Pro, Cya, Bct, Asc, Act, Str	43	1.23	2421	1.83	1.7
REC-HTH18	AraC	PF12833	DNA-binding	1xs9	Fir, Pro, Cya, Bct	43	1.23	1839	1.67	2.34
REC-HDOD	HDOD	PF08668	Phosphohydrolases	1vqr	Ver, Pro, Def	34	0.98	131	0.13	25.9
REC-Sigma54_activat	Sigma54	PF00158	Transcriptional control	1ny5	Ver, Pro, Cni, Bct, Aqu, Str, Nit	23	0.66	856	0.82	2.6
BetR-REC	BetR	PF08667	DNA-binding	na	Pro	20	0.57	84	0.08	23.8
REC-Myb_DNA-binding	Myb	PF00249	DNA-binding	2uxn	Chl, Str	18	0.52	149	0.13	12.0
REC-PPDK_N	PPDK	PF01326	Pyruvate phosphate diKinases	2ols	Bct, Aci, Spi	18	0.52	18	0.02	100.0
REC-GGDEF-EAL	GGDEF	PF00990 PF00563	Cyclic di-GMP signaling	1w25; 4j40	Gem, Pro, Cya, Bct, Act, Clf, Eur	18	0.52	225	0.21	8.0
REC-HisKA-HATPase_c	Histidine kinase	PF00512 PF02518	Histidine kinases	2c2a	Fir, Pro, Cya, Bct, Act, Clf, Eur	18	0.52	2421	1.83	0.7
REC-YcbB	YcbB (GlnL)	PF08664	DNA-binding	na	Fir	16	0.46	255	0.24	6.2
REC-SpoIIE	PP2C (SpoIIE)	PF07228	Protein phosphatase (Ser, Thr)	3eq2	Pla, Fir, Fus, Pro, Bct, Act	15	0.43	496	0.45	3.0
REC-HD5	HD5	PF13487	Phosphohydrolases	3tmb	Fir, Pro, Bct	15	0.43	431	0.43	3.4

REC-DUF3685	DUF3685	PF12452	Unknown function	na	Cya	12	0.34	28	0.03	42.8
REC-CbiA	Colbamin	PF01656	Colbamin biosynthesis	1cbu	Fir, Pro, Act	11	0.32	24	0.02	45.8

[a]Only for output domain architectures that constitute greater or equal to 2% of the total ALR sequences identified. Table and annotations are based on ref. 18.

[b]Architecture of the ED

[c]Common name given to the ED family

[d]Pfam identification code

[e]Known function of the ED family

[f]ID code of a representative PDB file from the RCSB Protein Databank

[g]Classes that have at least one of the ED in question

[h]Total number of ALR sequences that have the ED in question

[i]Percentage contribution of the ED in question to the total ALR database

[j]Total number of REC (with ALR) sequences that have the ED in question

[k]Percentage contribution of the ED in question to the total REC database (REC plus ALR sequences).

[l]Percentage contribution of the ED in question to the total ALR database (only ALR sequences).
