# Supplementary material for: The Aspartate-Less Receiver (ALR) Domains: Distribution, Structure and Function
Source: PLoS Pathog. 2015 Apr 13;11(4):e1004795. doi: 10.1371/journal.ppat.1004795 (PMC4395418; doi:10.1371/journal.ppat.1004795)
Supplement: S3 Table — (PDF) [file ppat.1004795.s010.pdf]

Table S3. Deposited ALR Structures.

ALR	PDB code	Bacterial Species	Function	Domains[a]	Y/T Coupling Residues	Y/T Rotomer	Acidic Triad Residues[b]	Oligo-meric State[c]	Ref.[d]
RitR	4LZL	*Streptococcus pneumoniae*	iron/redox regulation, pathogenesis	REC-wHTH	Y/D	out/in	E,K,N	M	This work
AmiR	1QO0	*Pseudomonas aeruginosa*	transcriptional termination	REC-ANTAR	V/V	in	P,P,S	D	[17]
ChxR	3Q7R	*Chlamydia trachomatis*	development, pathogenesis	REC-wHTH	Y/L	out	S,E,E	D	[27]
HP1043	2PLN	*Helicobacter pylori*	growth *in vitro*	REC-wHTH	Y/S	in	E,K,S	D	[25]
FrzS	2I6F	*Myxococcus xanthus*	polar localization, motility	REC-Coiled-Coil-GluZincin	Y/T	in	E,S,A	T*,H*	[28]
KaiA	1M2E	*Synechococcus elongatus*	circadian clock regulation	REC-PP2C phosphatase	H/G	in	V,E,V	M	[29]
n/a	2B4A	*Bacillus halodurans*	unknown	REC-wHTH	Y/T	in	E,D,S	M	Unch.
n/a	2QZJ	*Clostridium difficile*	unknown	REC	Y/T	in	D,G,E	D	Sugadev *et al.* Unch.
n/a	2ZAY	*Desulfuromonas acetoxidans*	unknown	Type II Secretion system protein-REC	F/S	in	D,T,E	D	Fedorov *et al.* Unch.

n/a	3HV2	*Pseudomonas fluorescens*	unknown	REC-HD	Y/T	in	D,S,A	D	Patskovsky *et al*. Unch.
n/a	3KTO	*Pseudo-alteromonas atlantica*	unknown	REC	F/A	in	D,H,E	T*	Damodharan *et al.* Unch.

[a]wHTH, winged helix-turn-helix; ANTAR, RNA binding domain; GluZincin, aminopeptidase domain; HD, phosphohydrolase domain.

[b]Acidic triad residue in their order of appearance (N-C terminus: Acidic triad-1, acidic triad-2, changed phospho-accepting aspartate coordinate).

[c]M, monomer; D, dimer; T, trimer; H, hexamer.

[d]Unch. Uncharacterized protein or soon to be published. See entry in the RCSB PDB protein databank.

[*]Dimer is not facilitated through the canonical REC alpha4-beta4-alpha5 interface.
