# Supplementary material for: The Aspartate-Less Receiver (ALR) Domains: Distribution, Structure and Function
Source: PLoS Pathog. 2015 Apr 13;11(4):e1004795. doi: 10.1371/journal.ppat.1004795 (PMC4395418; doi:10.1371/journal.ppat.1004795)
Supplement: S4 Table — (PDF) [file ppat.1004795.s011.pdf]

Table S4. Oligonucleotides Used in This Study

Primer Name[a]	5'-3' Primer Sequence[b]	Description[c]
RitR REC F1	***AGGT***ATGGGGAAACGGATTTTATTACTTGAG	SUMO-RitR REC/FL
RitR REC F2	ATGGGGAAACGGATTTTATTACTTGAG	SUMO-RitR REC/FL
RitR REC R1	***CTAG***CCTTCTTGCATGGTATATCCAACACCAC	SUMO-RitR REC
RitR REC R2	CCTTCTTGCATGGTATATCCAACACCAC	SUMO-RitR REC
RitR FL R1	***CTAG***CCTTCTTGCATGGTATATCCAACACCAC	SUMO-RitR FL
RitR FL R2	CCTTCTTGCATGGTATATCCAACACCAC	SUMO-RitR FL
BS2-HEX-F	***Z***-ATTTTATTGACAGATTAGAAAAATAATGTTACA	HEX Piu Promoter BS2
BS2-R	TGTAACATTATTTTTCTAATCTGTCAATAAAAT	Piu Promoter BS2
RitR L86A F	GATCATTGGGAAGAC***GCG***CAAGAAGAGCTGG	Leu86 > Ala mutation
RitR L86A R	CCAGCTCTTCTTG***CGC***GTCTTCCCAATGATC	Leu86 > Ala mutation
RitR L90A F	GACTTGCAAGAAGAG***GCG***GAAGTTGTTCAGCG	Leu90> Ala mutation
RitR L90A R	CGCTGAACAACTTC***CGC***CTCTTCTTGCAAGTC	Leu90 > Ala mutation
RitR V93A F	CAAGAAGAGCTGGAAGTT***GCG***CAGCGTTTTGCAGTTTC	Val93 > Ala mutation
RitR V93A R	GAAACTGCAAAACGCTG***CGC***AACTTCCAGCTCTTCTTG	Val93 > Ala mutation
RitR L86A/V93A F	GGAAGAC***GCG***CAAGAAGAGCTGGAAGTT***GCG***CAGCGTTTTG	Leu86 > Ala / Val93 > Ala double mutation
RitR L86A/V93A R	CAAAACGCTG***CGC***AACTTCCAGCTCTTCTTG***CGC***GTCTTCC	Leu86 > Ala / Val93 > Ala double mutation
RitR F96G F	CTGGAAGTTGTTCAGCGT***GGT***GCAGTTTCATACATCTATAAG	Phe96 > Gly mutation
RitR F96G R	CTTATAGATGTATGAAACTGC***ACC***ACGCTGAACAACTTCCAG	Phe96 > Gly mutation
RitR Y100A F	CAGCGTTTTGCAGTTTCA***GCC***ATCTATAAGCCAGTCC	Tyr100 > Ala mutation
RitR Y100A R	GGACTGGCTTATAGAT***GGC***TGAAACTGCAAAACGCTG	Tyr100 > Ala mutation
RitR Y102F F	GTTTTGCAGTTTCATACATC***GCG***AAGCCAGTCCTTATCG	Tyr102 > Phe mutation
RitR Y102F R	CGATAAGGACTGGCTT***CGC***GATGTATGAAACTGCAAAAC	Tyr102 > Phe mutation
RitR D81A F	GCCTCAGTCATCATGATTTTA***GCT***CATTGGGAAGACTTGCAAGAAG	Asp81 > Ala mutation
RitR D81A R	CTTCTTGCAAGTCTTCCCAATG***AGC***TAAAATCATGATGACTGAGGC	Asp81 > Ala mutation
RitR N53A F	GACAGACTATGATTTGATTTTATTG***GCC***GTTAATCTGGGAGATATG	Asn53 > Ala mutation
RitR N53A R	CATATCTCCCAGATTAAC***GGC***CAATAAAATCAAATCATAGTCTGTC	Asn53 > Ala mutation
RitR N53D F	GACAGACTATGATTTGATTTTATTG***GAC***GTTAATCTGGGAGATATG	Asn53 > Asp mutation
RitR N53D R	CATATCTCCCAGATTAAC***GTC***CAATAAAATCAAATCATAGTCTGTC	Asn53 > Asp mutation
RitR K10D F	GGAAACGGATTTTATTACTTGAG***GAC***GAACGAAATCTAGCTC	Lys10 > Asp mutation
RitR K10D R	GAGCTAGATTTCGTTC***GTC***CTCAAGTAATAAAATCCGTTTCC	Lys10 > Asp mutation
RitR Kan check-F1	5'-GGTGTACCATTGTCACTGATTACTG-3'	*5' ritR flank*
RitR Kan check-R1	5'-CAATTCCGGTGATATTCTCATTTTAGCC-3'	*5' ritR flank*
RitR Kan check-F2	5'-GGGATCAAGCCTGATTGGGAG-3'	*3' ritR flank*
RitR Kan check-R2	5'-CCTAGCTTATTTAAATAATACCAATTGCCG-3'	*3' ritR flank*
erm-cassette-F	5'-GGTGTACCATTGTCACTGATTACTG-3'	*5' erm cassette*
erm-cassette-R	5'-CCTAGCTTATTTAAATAATACCAATTGCCG-3'	*3' erm cassette*
5- FL-check-F	5'-CAATGGTATTGAGTACGGTGATATGC-3'	*5' ritR flank*
5'-FL-check-R	5'-CTTTCTCAAGTAATAAAATCCGTTTCCC-3'	*5' ritR flank*
3'-FL-check-F	5'-CCGTTACTTATGAGCAAGTATTGTC-3'	*3' ritR flank*
3'-FL-check-R	5'-CAAGGTATTGCCATCCTGTTTTC-3'	*3' ritR flank*
Ppiu-F1	5'-*AATT*CTGAAGTTGGCATTCAAAAAATCATGTTC-3'	*Piu promoter*
Ppiu-F2	5'-CTGAAGTTGGCATTCAAAAAATCATGTTC-3'	*Piu promoter*
Ppiu(1+2+3)-R1	5'-*GATCC*GACTAGGAGTAGAAGTAAGCC-3'	*Piu promoter*
Ppiu(1+2+3)-R2	5'-CGACTAGGAGTAGAAGTAAGCC-3'	*Piu promoter*
PP2-tet-F	5'-CCTAATCGGAAAGGTTTTCAATCCC-3'	*PP2 tet gene*
PP2-tet-R	5'-CCGATAACGATACCAAGGATGAAAC-3'	*PP2 tet gene*
PP2-bga-F	5'-GTCTTGGTTGGAACCAAACCAG-3'	*PP2 bga gene*
PP2-bga-R	5'-GTTTCAATCTACTATACAATAAGAGAACG-3'	*PP2 bga gene*
PP2-seq-F	5'-GCATGCATCGGTACCTGCG-3'	*PP2 plasmid*
PP2-seq-R	5'-GTAAAACGACGGGATCAAGATGTTTC-3'	*PP2 plasmid*

[a]F, forward primer; R, reverse primer; HEX, 5' Hexachlorofluoroscein label; BS2, *piu* promoter Binding Site 2

[b]Z, HEX moiety; Bold italicized underlined and **red**, *Bsa*I restriction site; **green**, mutated Gate codon; **blue**, mutated catalytic domain codon.

[c]REC, Receiver Domain; FL, Full-length protein; >, Mutated to the residue that follows.
